# Zinc Finger Proteins in Head and Neck Squamous Cell Carcinomas: ZNF540 May Serve as a Biomarker

**DOI:** 10.3390/curroncol29120779

**Published:** 2022-12-16

**Authors:** Joanna Sobocińska, Joanna Nowakowska, Sara Molenda, Anna Olechnowicz, Kacper Guglas, Joanna Kozłowska-Masłoń, Urszula Kazimierczak, Marta Machnik, Urszula Oleksiewicz, Anna Teresiak, Katarzyna Lamperska, Tomasz Kolenda

**Affiliations:** 1 Laboratory of Cancer Genetics, Greater Poland Cancer Center, Garbary 15, 61-866 Poznan, Poland; 2 Research and Implementation Unit, Greater Poland Cancer Center, Garbary 15, 61-866 Poznan, Poland; 3 Department of Cancer Immunology, Chair of Medical Biotechnology, Poznan University of Medical Sciences, 8 Rokietnicka Street, 60-806 Poznan, Poland; 4 Molecular and Cell Biology Unit, Poznan University of Medical Sciences, 60-572 Poznan, Poland; 5 Department of Histology and Embryology, Poznan University of Medical Sciences, Święcickiego 6 Street, 60-781 Poznan, Poland; 6 Postgraduate School of Molecular Medicine, Medical University of Warsaw, Zwirki and Wigury Street 61, 02-091 Warsaw, Poland; 7 Institute of Human Biology and Evolution, Faculty of Biology, Adam Mickiewicz University, Uniwersytetu Poznańskiego 6, 61-614 Poznan, Poland; 8 Department of Diagnostics and Cancer Immunology, Greater Poland Cancer Center, Garbary 15, 61-866 Poznan, Poland

**Keywords:** HNSCC, *HPV*, KRAB-ZNF, ZNF, biomarkers, TCGA, epigenetic

## Abstract

Head and neck squamous cell carcinoma (HNSCC) is one of the ten most common cancers. Most cancer cases originate from alcohol and tobacco consumption. However, studies have demonstrated that human papillomavirus (*HPV*) infection, particularly *HPV-16*, may also significantly influence disease progression. The KRAB-ZNF family of genes is involved in epigenetic suppression, and its involvement in carcinogenesis is the subject of extensive studies. The available literature data demonstrate that they may play different roles, both as tumor suppressors and oncogenes. In this study, six ZNF genes, *ZFP28*, *ZNF132*, *ZNF418*, *ZNF426*, *ZNF540*, and *ZNF880*, were tested using several in silico approaches based on the TCGA and GEO datasets. Our analyses indicate that the expression of the analyzed ZNFs was significantly downregulated in tumor tissues and depended on tumor localization. The expression levels of ZNFs differed between *HPV*-positive vs. *HPV*-negative patients depending on the clinical-pathological parameters. More specifically, the patients with higher levels of *ZNF418* and *ZNF540* showed better survival rates than those with a lower expression. In addition, the level of *ZNF540* expression in *HPV*-positive (*HPV(+)*) patients was higher than in *HPV*-negative (*HPV(−)*) patients (*p* < 0.0001) and was associated with better overall survival (OS). In conclusion, we demonstrate that *ZNF540* expression highly correlates with *HPV* infection, which renders *ZNF540* a potential biomarker for HNSCC prognosis and treatment.

## 1. Introduction

Head and neck squamous cell carcinoma (HNSCC) contributed to the death of 450,000 people worldwide in 2018, which makes it the seventh most severe cancer. HNSCC is mainly associated with tobacco and alcohol abuse. However, human papillomavirus (*HPV*) infection, mostly with *HPV-16*, also appears as a crucial etiologic factor in HNSCC development [1,2]. The characteristics of HNSCC are a poor response to treatment and high mortality, where only about 50–60% of patients reach the 5-year survival rate. Thus, there is an urgent need to develop novel, more effective, personalized therapies and specific prognostic biomarkers which are based on genes with protein-coding and non-coding abilities [3,4,5,6]. However, knowledge of the exact molecular mechanisms driving HNSCC is still limited.

Zinc finger proteins (ZNFs) constitute the most numerous family of sequence-specific DNA-binding proteins encoded by 2% of human genes. They bind to their target DNA sequences through the zinc finger domain [7] and exert various functions, including transcriptional regulation, signal transduction, or protection against DNA double-strand breaks [8,9,10]. They may interact with DNA sequences, RNAs, proteins, and post-translational modifications [8,9,10]. ZNFs are divided into several subgroups based on their structural conformation, and C2H2 ZNFs are the most common [7,11]. Besides various zinc finger motifs, the C2H2 class contains additional domains involved in gene expression or cellular localization—such as the KRAB (Krüppel-associated box), SCAN, or BTB/POZ domain [12,13]. Due to the considerable complexity within the ZNF family, little is known about the exact molecular function of most of its members. Of note, many KRAB-ZNFs were shown to play an essential role in carcinogenesis, acting as oncogenes, suppressors, or both, depending on the cancer type [14]. The association with tumor biology was already described for several ZNFs in various cancers, including melanoma [15], colorectal [16,17], renal [18], gastric [19], and esophageal cancers [20], or lung adenocarcinomas [13]. Numerous KRAB-ZNFs show altered expression in various tumors, e.g., HNSCC, as was demonstrated in the transcriptomic profiling based on the TCGA datasets [21]. Nevertheless, the contribution to biological processes and the potential diagnostic utility of specific ZNFs in HNSCC remain undefined. Moreover, there isstill no data on ZNFs’ involvement in head and neck cancers with *HPV* origin.

For this study, based on the preselection with the UALCAN database [22], we chose six ZNF genes: *ZFP28*, *ZNF132*, *ZNF418*, *ZNF426*, *ZNF540*, and *ZNF880*. In our previous analysis, these factors were shown to be downregulated in multiple tumor types, including HNSCC [21]. Moreover, *ZNF132* was reported to be epigenetically inactivated in laryngeal squamous cell carcinoma due to promoter hypermethylation [23,24]. In the same tumor type, *ZNF418* promoter methylation was demonstrated as a potent diagnostic factor distinguishing between high- and low-risk groups of patients [25]. To the best of our knowledge, no other report has been published to date describing the involvement of *ZFP28*, *ZNF132*, *ZNF418*, *ZNF426*, *ZNF540*, and *ZNF880* in HNSCC. Here, we hypothesize that these genes may be implicated in HNSCC biology and related *HPV* phenotypes. To test this hypothesis, we used the TCGA data and performed bioinformatics analyses of mRNA expression. We aimed to explore the correlation of ZNF expression with clinico-pathological parameters, their engagement in various cancer-associated processes, and their potential role as biomarkers in HNSCC.

## 2. Materials and Methods

**Figure 1:**
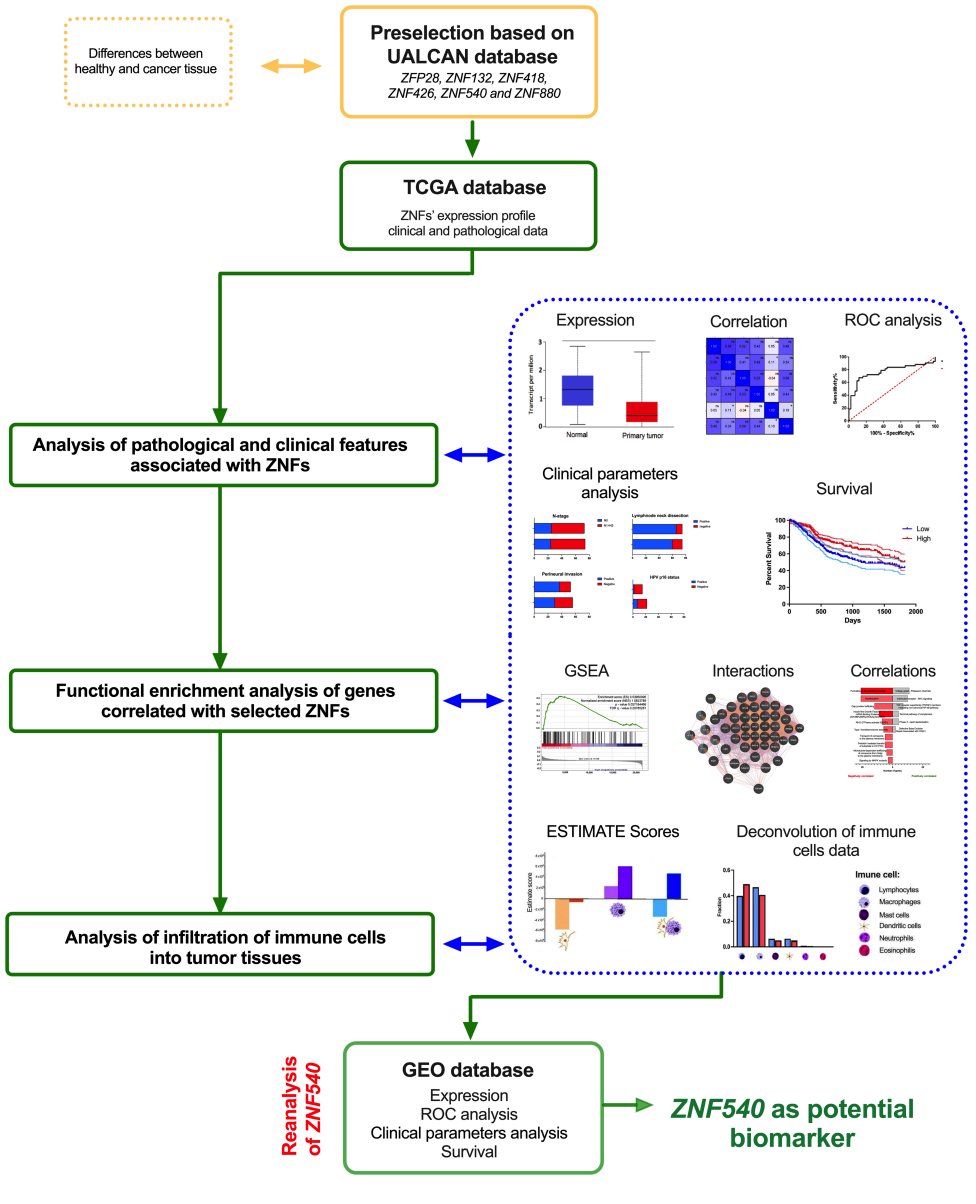
The main steps of the methodology and tools used in the presented study. UALCAN—The University of Alabama at Birmingham CANcer data analysis Portal; TCGA—The Cancer Genome Atlas; ROC—Receiver operating characteristic; ESTIMATE Scores—Estimation of Stromal and Immune cells in Malignant Tumor tissues using Expression data; GSEA—Gene Set Enrichment Analysis; GEO—Gene Expression Omnibus.

In our study, we analyzed six ZNFs preselected based on the UALCAN database: *ZFP28*, *ZNF132*, *ZNF418*, *ZNF426*, *ZNF540*, and *ZNF880*, using the RNA sequencing data downloaded from the TCGA [22]. The study’s main steps included: (i) analysis of pathological and clinical features associated with ZNFs, (ii) functional enrichment analysis of genes correlated with selected ZNFs, (iii) analysis of infiltration of immune cells into tumor tissues, and (iv) validation of the selected results. The main steps of the methodology used by us are presented in Figure 1.

### 2.1. TCGA Data

The TCGA expression data of *ZFP28*, *ZNF132*, *ZNF418*, *ZNF426*, *ZNF540*, and *ZNF880*, along with clinical data (International Classification of Diseases, Tenth Revision (ICD-10), World Health Organization (WHO)), were downloaded from the Santa Cruz University of California Data Set (Head and Neck Squamous Cell Carcinoma, TCGA, dataset: gene expression RNAseq—IlluminaHiSeq pancan-normalized; RNA expression pan-cancer-normalized log2(norm_count + 1)) and from the UALCAN database (http://ualcan.path.uab.edu/ (accessed on 10 November 2020)) [22].

### 2.2. Pathological and Clinical Analysis

The differences between healthy and cancer tissues for analyzed ZNFs were obtained from the UALCAN database. To determine whether the expression level of each transcript allowed us to distinguish healthy from cancer samples, we applied a receiver operating characteristic (ROC) analysis with an area under the curve (AUC) estimation in a group of 43 adjacent-matched healthy and neoplastic tissues. We performed the Spearman correlation test to assess the correlation between expression levels of analyzed ZNFs. Next, expression levels of ZNFs were checked depending on localizations in the oral cavity (*n* = 316), pharynx (*n* = 90), and larynx (*n* = 116).

Furthermore, we correlated the expression levels of *ZFP28*, *ZNF132*, *ZNF418*, *ZNF426*, *ZNF540*, and *ZNF880* genes with various clinical parameters, including age (<61 vs. >61), gender (female vs. male), alcohol history (positive vs. negative), smoking history (No/Ex vs. Yes), cancer stage (I + II vs. III + IV), T stage (T1 + T2 vs. T3 + T4), N stage (N0 + N1 vs. N2 + N3), cancer grade (G1 + G2 vs. G3 + G4), perineural invasion (positive vs. negative), lymph node neck invasion (positive vs. negative), angiolymphatic invasion (positive vs. negative), and *HPV p16* status (negative vs. positive) in all localizations of the HNSCC samples. The number of patient cases analyzed in the groups depended on the specific clinical parameters we present in Supplementary Table S1.

To determine the differences in overall survival (OS) and disease-free interval (DFI), the patients were divided into two groups depending on the expression level of the specified gene using the mean expression level as cut-off: *ZNF418* for OS: *n_Low_* = 271, *n_High_* = 250 and for DFI: *n_Low_* = 68, *n_High_* = 62; *ZNF540* for OS: *n_Low_* = 261, *n_High_* = 260 and for DFI: *n_Low_* = 55, *n_High_* = 75. The time of the observation was set as up to 5 years (1825 days).

### 2.3. Functional Enrichment Analysis of Genes Correlated with Selected ZNFs

Genes correlated with the analyzed transcripts were acquired from the cBioPortal for Cancer Genomics (www.cbioportal.org (accessed on 20 November 2020)). Positively (*R* > 0.3) and negatively (*R* < −0.3) correlated genes, according to the Spearman correlation, were used to study cellular involvement and interactions with a REACTOME database (https://reactome.org (accessed on 20 November 2020)). To determine statistically significant correlations, *p* < 0.05 was used for negative correlations with all analyzed transcripts and positive correlations with *ZFP28*, *ZNF132*, *ZNF418*, *ZNF426*, *ZNF540*, and *ZNF880*.

Gene Set Enrichment Analysis (GSEA) software version 4.1 (http://www.gsea-msigdb.org/gsea/index.jsp (accessed on 12 January 2021)) was used to analyze the functional enrichment for HSNCC patients divided into two groups with low and high expression levels of specific ZNFs, using the mean expression levels as a cut-off (the same as for survival analysis). The input file contained expression data from TCGA for 20,530 genes and 520 patients. The groups were compared in terms of Hallmark gene sets (H) and Oncogenic Signatures (C) from the MSigDB collection using an analysis of 1000 gene permutations for testing the significance of the specified gene set enrichment. A nominal *p*-value of *p* ≤ 0.05 and a false discovery rate (FDR) ≤ 0.25 were considered significant, as described previously [26].

### 2.4. Infiltration of Immune Cells into Tumor Tissues

Analysis of the Immune and ESTIMATE scores (Estimation of STromal and Immune cells in MAlignant Tumor tissues using Expression data) was downloaded from https://bioinformatics.mdanderson.org/estimate/disease.html (accessed on 12 December 2020). These scores were used to define the infiltration of immune cells into tumor tissues and to infer tumor purity. The subpopulations of specific immune cells were estimated using supporting data presented by Thorsson et al. [27] and analyzed as described previously [26].

### 2.5. Statistical Analysis

All statistical analyses were performed using GraphPad Prism 6, 8, and 9 (GraphPad, San Diego, CA, USA). For two-group analysis, the *t*-test or Mann–Whitney U test were used for measuring *ZFP28*, *ZNF132*, *ZNF418*, *ZNF426*, *ZNF540*, and *ZNF880* levels and gene expression depending on the Shapiro–Wilk normality test. Next, the expression levels of ZNFs were compared between tumor localizations using the one-way ANOVA and Tukey’s multiple comparisons test or Dunn’s multiple comparisons test. Receiver operating characteristic (ROC) curve analysis of *ZFP28*, *ZNF132*, *ZNF418*, *ZNF426*, *ZNF540*, and *ZNF880* expression was used to compare adjacent normal and cancerous tissues obtained for 43 patients, and the AUC (Area Under Curve) with a 95% Confidence Interval (CI) was calculated.

For OS and DFI prognosis, the Log-Rank (Mantel–Cox) and Gehan–Breslow–Wilcoxon tests were used, and a 95% CI ratio was calculated. A heatmap was generated using MORPHEUS, an online visualization tool (https://software.broadinstitute.org/Morpheus (accessed on 20 January 2021)). For all of the analyses, *p* < 0.05 was indicated as statistically significant.

### 2.6. Validation of the Results

To validate the obtained results from the TCGA database, we used the Gene Expression Omnibus (GEO) data repository, with GSE65858 [28] set for HNSCC samples. The *ZNF540* expression was compared between *HPV(−)* and *HPV(+)* (*n* = 176 vs. 94), *HPV(−)* vs. *HPV-16* vs. other types of *HPV* (*n* = 196 vs. 61 vs. 13), as well as between active *HPV(+)* infection (DNA+/RNA+) vs. inactive *HPV(+)* infection (DNA+/RNA−) (*n* = 35 vs. 19). The ROC analysis was applied to the assessment of the *ZNF540* expression level to discriminate the activity of viral infection (active vs. inactive; *n* = 35 vs. 19). Next, we evaluated the correlation between the expression levels of *ZNF540* and various clinicopathological parameters such as age (<60 vs. >60; *n* = 157 vs. 113), gender (female vs. male; *n* = 47 vs. 223), smoking history (yes vs. no; *n* = 222 vs. 48), alcohol consumption (yes vs. no; *n* = 239 vs. 31), disease stage (I–II vs. III–IV; *n* = 55 vs. 215), T stage (T1 + T2 vs. T3 + T4; *n* = 115 vs. 155), N stage (N0 vs. N1 + N2 + N3; *n* = 94 vs. 176), cancer molecular clusters (atypical IR1, basal 4, classical 2, mesenchymal 4; *n* = 73 vs. 84 vs. 30 vs. 83, respectively), and localizations (oral cavity, hypopharynx, larynx, oropharynx; *n* = 83 vs. 33 vs. 48 vs. 102, respectively). Finally, we analyzed patients’ OS in *HPV(−)* and *HPV(+)* groups (*n* = 196 vs. 73), and next in groups divided based on *ZNF540* expression levels (using the mean of expression in analyzed groups as a cut-off): all patients (*n_Low_* = 176, *n_High_* = 94), only *HPV(−)* (*n_Low_* = 123, *n_High_* = 72), and only *HPV(+)* (*n_Low_* = 35, *n_High_* = 25) groups. The statistical analysis was performed as described above.

## 3. Results

The ZNFs are downregulated in HNSCC and show a high potential to distinguish normal from cancer tissues.

**Figure 2:**
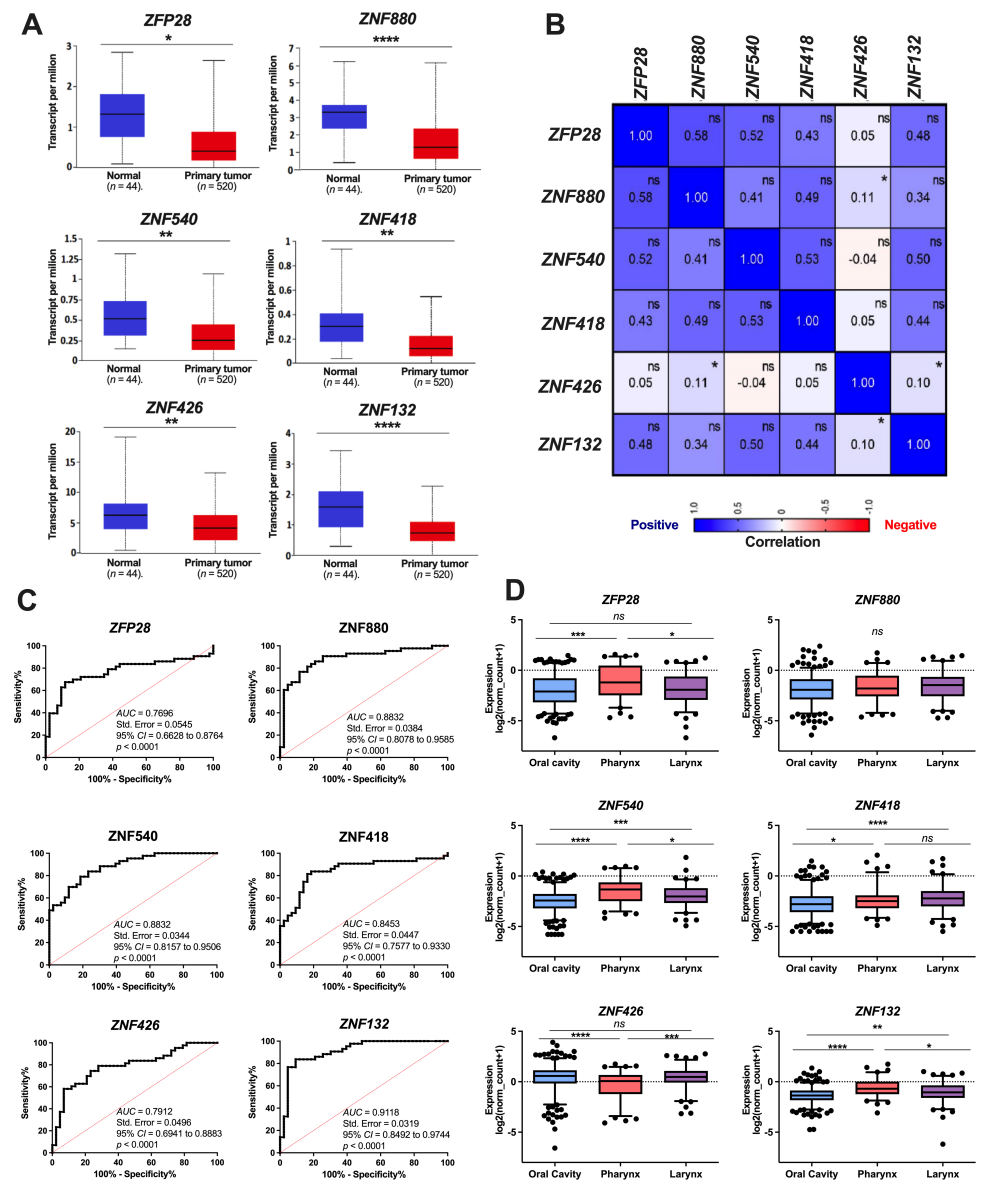
(**A**) The expression levels of *ZFP28*, *ZNF132*, *ZNF418*, *ZNF426*, *ZNF540*, and *ZNF880* in HNSCC patients. Expression in normal (*n* = 44) and cancer tissues (*n* = 520). Graphs were obtained from the UALCAN database and modified. (**B**) The Spearman correlation between all analyzed ZNFs. R values are provided in each square of the heatmap. (**C**) Receiver operating characteristic (ROC) curve analysis of *ZFP28*, *ZNF132*, *ZNF418*, *ZNF426*, *ZNF540*, and *ZNF880* expression comparing adjacent normal and cancerous tissues obtained from 43 patients. (**D**) Expression levels of *ZFP28*, *ZNF132*, *ZNF418*, *ZNF426*, *ZNF540*, and *ZNF880* depending on the cancer localization in the oral cavity (*n* = 316), pharynx (*n* = 90), and larynx (*n* = 116) in HNSCC patients. One-way ANOVA was used to assess the statistical significance. Graphs with box and whiskers present 5–95 percentile; CI—confidence interval; ns—not significant; * *p* ≤ 0.05; ** *p* ≤ 0.01; *** *p* ≤ 0.001; **** *p* ≤ 0.0001 are considered as significant.

Based on the UALCAN database, we observed a significant downregulation of *ZFP28*, *ZNF132*, *ZNF418*, *ZNF426*, *ZNF540*, and *ZNF880* expression levels in primary tumor tissues compared to normal tissues (Figure 2A). Moreover, the data indicate a low number of positive correlations between the expression of: *ZNF426* and *ZNF880* (R = 0.11, *p =* 0.0093), as well as *ZNF426* and *ZNF132* (*R* = 0.10, *p* = 0.025) genes. For the rest of the analyzed ZNFs, no significant (*p >* 0.05) correlations were indicated (Figure 2B).

Next, we applied the ROC curve test to assess the potential of the analyzed ZNFs to discriminate between cancer and healthy tissues. To this end, we utilized paired normal and cancer tissues obtained from 43 HNSCC patients. The data indicate highly sensitive and specific discriminatory abilities for all six ZNFs, with the AUC ranging between 0.77 and 0.91 (*p* < 0.0001) (Figure 2C).

### 3.1. Expression of the ZNFs Depends on the Tumor Localization and Clinical-Pathological Parameters

Next, the ZNFs expression was analyzed in the HNSCC tissues obtained from various tumor localizations, including the oral cavity, larynx, and pharynx. Significant differences were observed in the expression level of *ZFP28*, *ZNF132*, *ZNF418*, *ZNF426*, and *ZNF540* between the oral cavity and pharynx localizations. The expressions of these factors were downregulated in the oral cavity, except for *ZNF426*, whose expression increased in the same localization. Moreover, we observed a significant difference in the expression of *ZFP28*, *ZNF132*, *ZNF426*, and *ZNF540* between the pharynx vs. larynx localizations, and *ZNF132*, *ZNF418*, and *ZNF540* in the oral cavity compared to the larynx. The expression levels of *ZFP28* and *ZNF426* were at the same level for the larynx localization and oral cavity. Moreover, the expression levels of *ZNF418*, *ZNF540*, and *ZNF132* were more similar between tumors located in the larynx and pharynx than the oral cavity. No significant differences were observed in *ZNF880* expression among the three cancer localizations (Figure 2D).

The expression levels of *ZFP28*, *ZNF132*, *ZNF418*, *ZNF426*, *ZNF540*, and *ZNF880* were also investigated in relation to the clinical-pathological parameters (Table 1). The analysis demonstrated that the expression of *ZFP28*, *ZNF132*, *ZNF418*, and *ZNF540* is significantly lower in patients over 60 years of age. Moreover, a lower transcription of *ZNF132*, *ZNF426*, and *ZNF540* was noticed in women compared to men. The two important carcinogenic factors, namely, alcohol consumption and smoking, were positively associated with higher *ZFP28* expression (*p* = 0.0480) and the elevated expression of *ZFP28*, *ZNF132*, *ZNF418*, and *ZNF880*, respectively.

Next, we correlated the expression of selected ZNFs with the TNM classification. In patients with higher T stages (3 and 4), the expression level of *ZFP28*, *ZNF132*, and *ZNF540* weresignificantly lower than in patients with a less advanced T stage. In the patients with stages N2 and N3, we observed an increased expression of *ZNF426* (*p =* 0.0012) and a decreased level of *ZNF132* (*p =* 0.0064) compared to the patients with N0 and N1. Moreover, in the group of patients with lymph node neck dissection, we noticed lower expressions of *ZFP28*, *ZNF132*, and *ZNF540*. Furthermore, in patients with grade 1 and 2 compared to the group with grade 3 and 4, the expression levels of *ZFP28*, *ZNF132*, *ZNF418*, and *ZNF540* were reduced, while that of *ZNF426* was increased. We also observed that patients with perineural invasion had a significantly decreased level of *ZNF540*, whereas patients with angiolymphatic invasion had lower levels of *ZNF426* and higher levels of *ZNF418* and *ZNF540*.

We associated the *HPV p16* status with the expression levels of ZNFs. We found that the lower level of *ZNF426* and the increased levels of *ZNF132* and *ZNF540* were characteristic for *HPV(+)* HNSCC patients. All results are presented in Table 1.

Finally, based on hierarchical clustering for the expression levels of ZNFs depending on clinical-pathological parameters, we observed that the direction of the changes in the expression levels of *ZNF540* and *ZNF132* was very similar in comparison to other ZNFs. Moreover, the expression levels of *ZNF426* were distinct compared to the rest of the analyzed genes for all clinical-pathological parameters (Supplementary Figure S1).

### 3.2. Patients with Low ZNF418 and ZNF540 Expression Display Shorter Overall Survival

**Figure 3:**
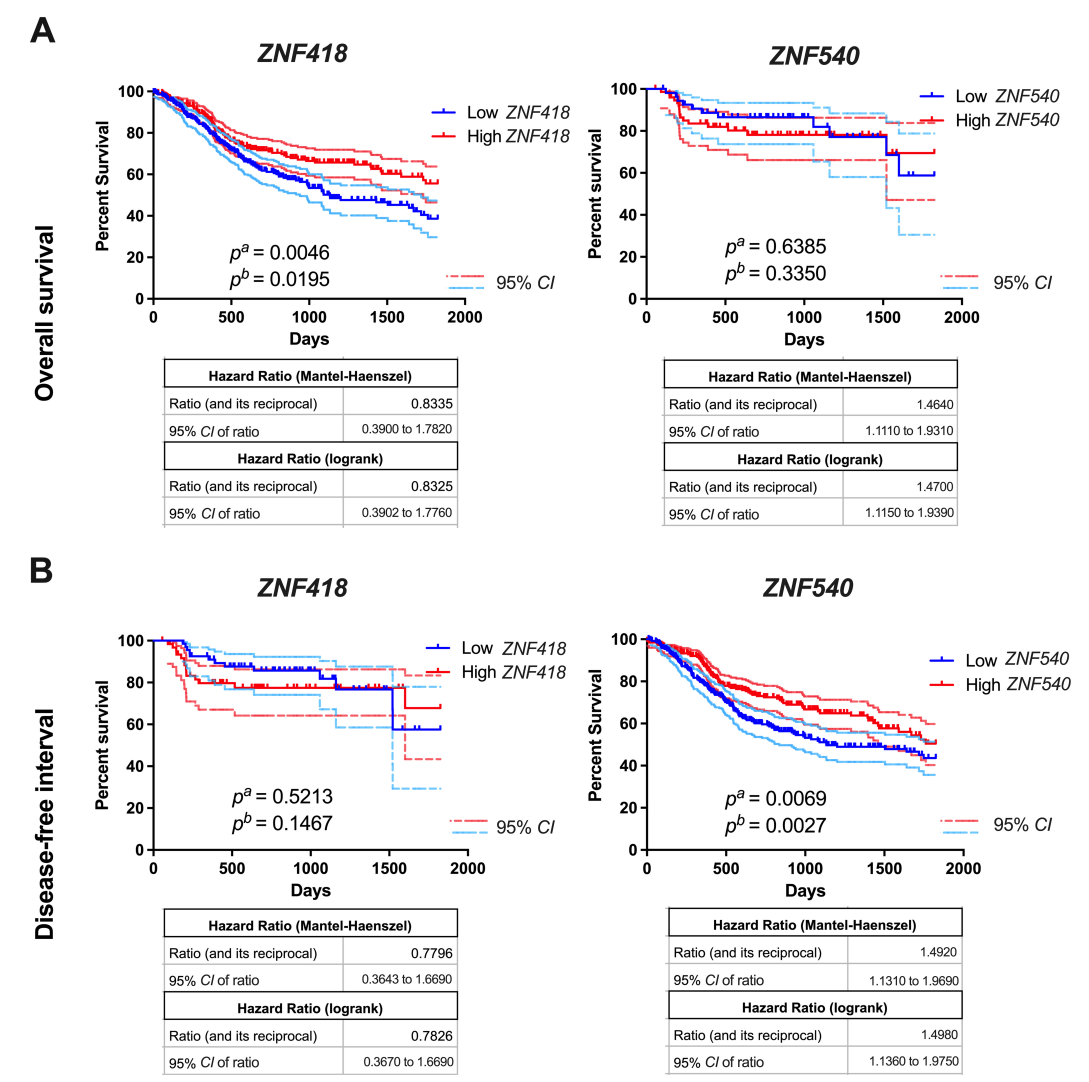
Overall survival (OS) (**A**) and disease-free interval (DFI) (**B**) of HNSCC patients depending on the *ZNF418* (for OS: *n_Low_* = 271, *n_High_* = 250; for DFI: *n_Low_* = 68, *n_High_* = 62) and *ZNF540* (for OS: *n_Low_* = 261, *n_High_* = 260; for DFI: *n_Low_* = 55, *n_High_* = 75) expression levels. The results are presented for 5 years of observation with 95% CI marked as lighter lines; the low and high subgroups of patients were divided based on the mean of expression. *n*—number of cases; CI—confidence interval; *p^a^* —Long–rank (Mantel–Cox) test; *p^b^*—Gehan–Breslow–Wilcoxon test; *p* < 0.05 is considered significant.

We further tested whether the expression of selected ZNFs may correlate with HNSCC patient outcome. To this end, we divided the patient cohort into two groups (high and low expression of each ZNF), with the mean expression used as a cut-off. We focused on the disease-free interval (DFI) and overall survival (OS). In the case of *ZFP28*, *ZNF132*, *ZNF426*, and *ZNF880*, no differences for OS or DFI were observed (*p* > 0.05). Moreover, there were no significant differences in DFI for *ZNF418* and *ZNF540*. However, the OS of patients significantly differed. A low expression of *ZNF418* and *ZNF540* was associated with worse OS compared to the increased expression. The results are presented in Figure 3 and Supplementary Figure S2.

### 3.3. Expression of the ZNFs Is Connected with Critical/Essential Cellular Processes and Pathways

Next, the genes with negative and positive correlations identified via the cBioPortal domain (Spearman correlation: *R* < −0.3 and *R* > 0.3) were analyzed using the REACTOME online tool. We concentrated mainly on the genes associated with *ZNF418* and *ZNF540*, as both factors showed a linkage to the patients’ survival (Figure 3). The correlated genes were classified into cellular processes and pathways. The studied ZNFs showed positive and negative correlations with various processes. The pathway that was negatively correlated with *ZNF418* and *ZNF540* included the formation of the cornified envelope, the keratinization process, gap junction trafficking, transport of connexons to the plasma membrane, microtubule-dependent trafficking of connexons from Golgi to the plasma membrane, and prefoldin--mediated transfer of substrate to CCT/TriC. The genes negatively associated only with *ZNF418* were involved in gap junction assembly, as well as trafficking and regulation, recruitment of NuMA to mitotic centrosomes, and carboxyterminal post-translational modifications of tubulin. The genes negatively correlated exclusively with *ZNF540* were connected with insulin-like growth factor-2 mRNA-binding proteins (IGF2BPs/IMPs/VICKZs) binding RNA, RHO GTPases activate IQGAPs, type I hemidesmosome assembly and signaling by MAPK mutants. For positively correlated genes, only those associated with voltage-gated potassium channels were common for these two ZNFs. Moreover, in this group, for *ZNF418*, the processes involved in elastic fiber formation, defective B3GALTL causes Peters-plus syndrome (PpS), O-glycosylation of TSR domain-containing proteins, molecules associated with elastic fibers, collagen chain trimerization, cGMP effects, defective CHST6 causes MCDC1, defective B4GALT1 causes B4GALT1-CDG (CDG-2d), and defective ST3GAL3 causes MCT12 and EIEE15 were indicated.

**Figure 4:**
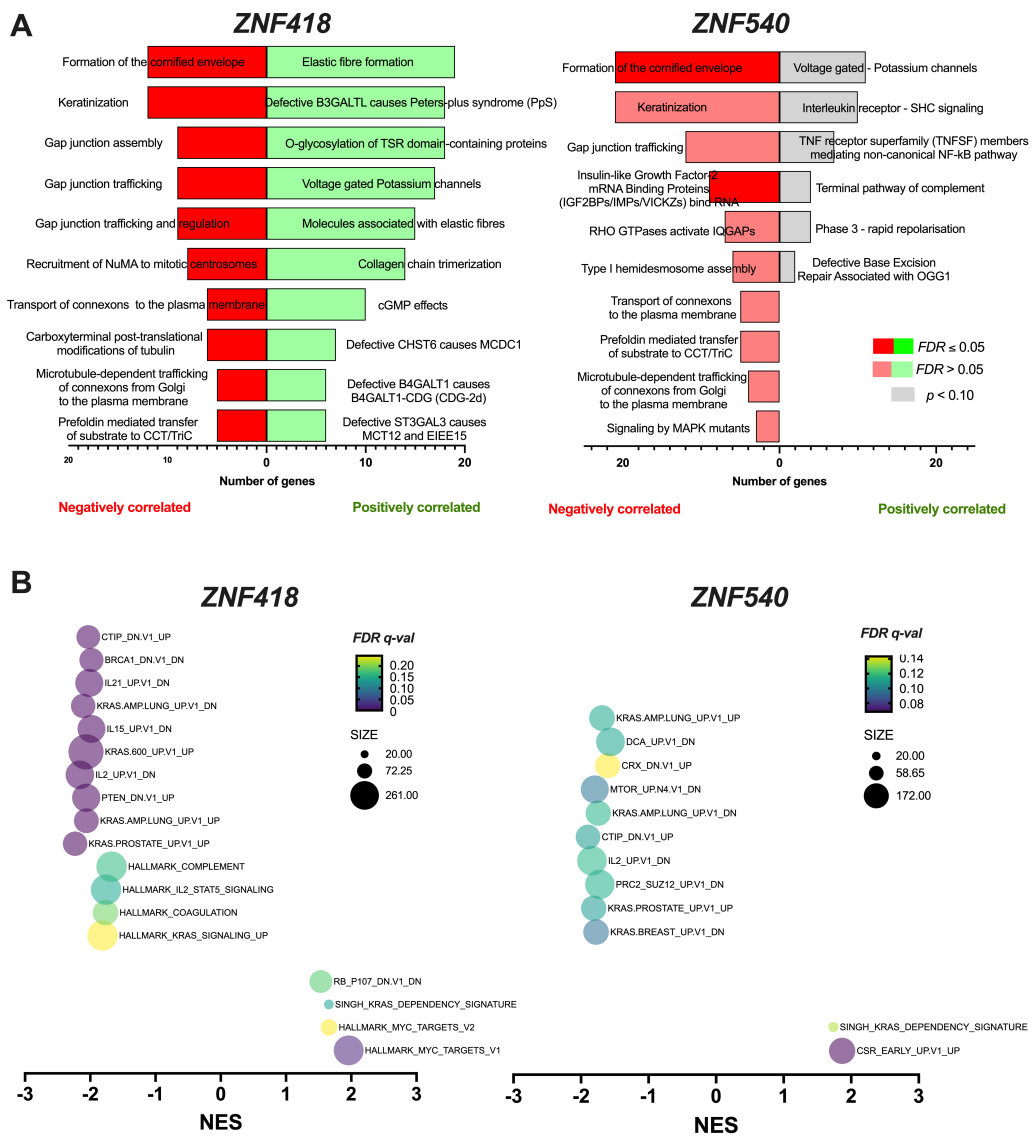
Association of *ZNF418* and *ZNF540* genes in cell processes and pathways in patients with HNSCC: (**A**) Classification of genes negatively and positively correlated with cellular processes and pathways based on the analysis of the REACTOME database. Only genes with Spearman’s correlation (*R* < −0.3 and *R* > 0.3, *p* < 0.05) were included in the analysis, and negatively correlated processes and pathways are marked in red, and positively in green. Results were marked in dark colors for FDR ≤ 0.05 and in light colors for FDR > 0.05; *p* < 0.05 was considered statistically significant and only for *p* < 0.10, it was indicated in gray. (**B**) GSEA of patients analyzed in groups of low vs. high expression of *ZNF418* (*n_Low_* = 271, *n_High_* = 250) and *ZNF540* (*n_Low_* = 261, *n_High_* = 260). Normalized enrichment scores for GSEA of MSigDB gene sets for the Hallmark gene set and oncogenic signatures. Only results with *p* ≤ 0.05 and FDR ≤ 0.25 were shown. NES (normalized enrichment score), *p*-val (nominal *p*-value), and FDR *q*-val (false discovery rate); *n*—number of cases.

The most significant number of genes (*n* = 53) was correlated with the pathway involved with *ZNF426*, namely, the regulation of the expression of SLITs and ROBOs. Moreover, several pathways associated with the studied ZNFs were connected to cancerogenesis, such as defective base excision repair associated with OGG1, which positively correlated with *ZNF540*; RAS signaling downstream of NF1 loss-of-function variants positively correlated with *ZNF426* and negatively with *ZNF880* and more. All data are presented in Figure 4A and in Supplementary Table S2.

Next, the GSEA (Gene Set Enrichment Analysis) was carried out and patients with a high and low expression of *ZNF418*, as well as *ZNF540*, were compared to show potential differences in processes and pathways. In the group of patients with a low expression of *ZNF418*, most upregulated genes are *MYC* targets (V1 and V2), genes connected with *KRAS*, genes downregulated in primary keratinocytes with knockdown of *RB1* and *RBL1* genes. Surprisingly, it was observed that, in the case of the patients with a high expression of *ZNF418*, 121 different processes and pathways were indicated. The most downregulated genes were connected with *KRAS* changes and *KRAS* signaling, coagulation, IL2*/STAT5* signaling, complement, genes associated with knockdown of *PTEN*, *BRCA1*, and *RBBP8*, and genes changed in cells after IL2 starvation and then stimulated by IL15 or IL21 (Figure 4B and Supplementary Table S3). In the case of patients with lower levels of *ZNF540*, it was indicated that the upregulation of genes was observed in foreskin fibroblasts in early response to serum starvation (CSR_EARLY_UP.V1_UP) and genes defining the *KRAS* dependency signature. For patients displaying higher levels of *ZNF540*, 48 different processes and pathways were indicated as changed, including genes connected with *KRAS*, genes changed in cells after knockdown of *SUZ12*, *RBBP8*, *CRX*, as well as genes changed after starvation and later stimulation by IL2, or genes changed in cells after treatment with *mTOR* pathway inhibitor or dichloroacetate (Figure 4B and Supplementary Table S3).

For *ZFP28*, *ZNF132*, *ZNF426*, and *ZNF880*, no associations with DFI and OS were observed, but some processes indicated in the GSEA displayed similarities with *ZNF418* and *ZNF540*. It was observed that the SING KRAS DEPENDENCY SIGNATURE process was changed for patients with lower levels of *ZFP28* and *ZNF880* similarly as for *ZNF418* and *ZNF540*, and in the case of HALLMARK_MYC_TARGETS_V1 for *ZFP28*, *ZNF418*, and *ZNF880*, as well as RB_P107_DN.V1_DN for *ZFP28* and *ZNF418*. Moreover, a similarity was observed for patients with a higher expression of *ZFP28*, *ZNF880*, *ZNF418*, and *ZNF540* for over 30 different processes and pathways connected with changes in GSEA’s gene sets. *ZFP28*, *ZNF132*, *ZNF880*, *ZNF418*, and *ZNF540* are connected with changes in IL2_UP.V1_DN. All data are summarized in Supplementary Table S3.

### 3.4. The Expression Levels of ZNFs Are Associated with the Tumor Immunological Profile in HNSCC Patients

**Figure 5:**
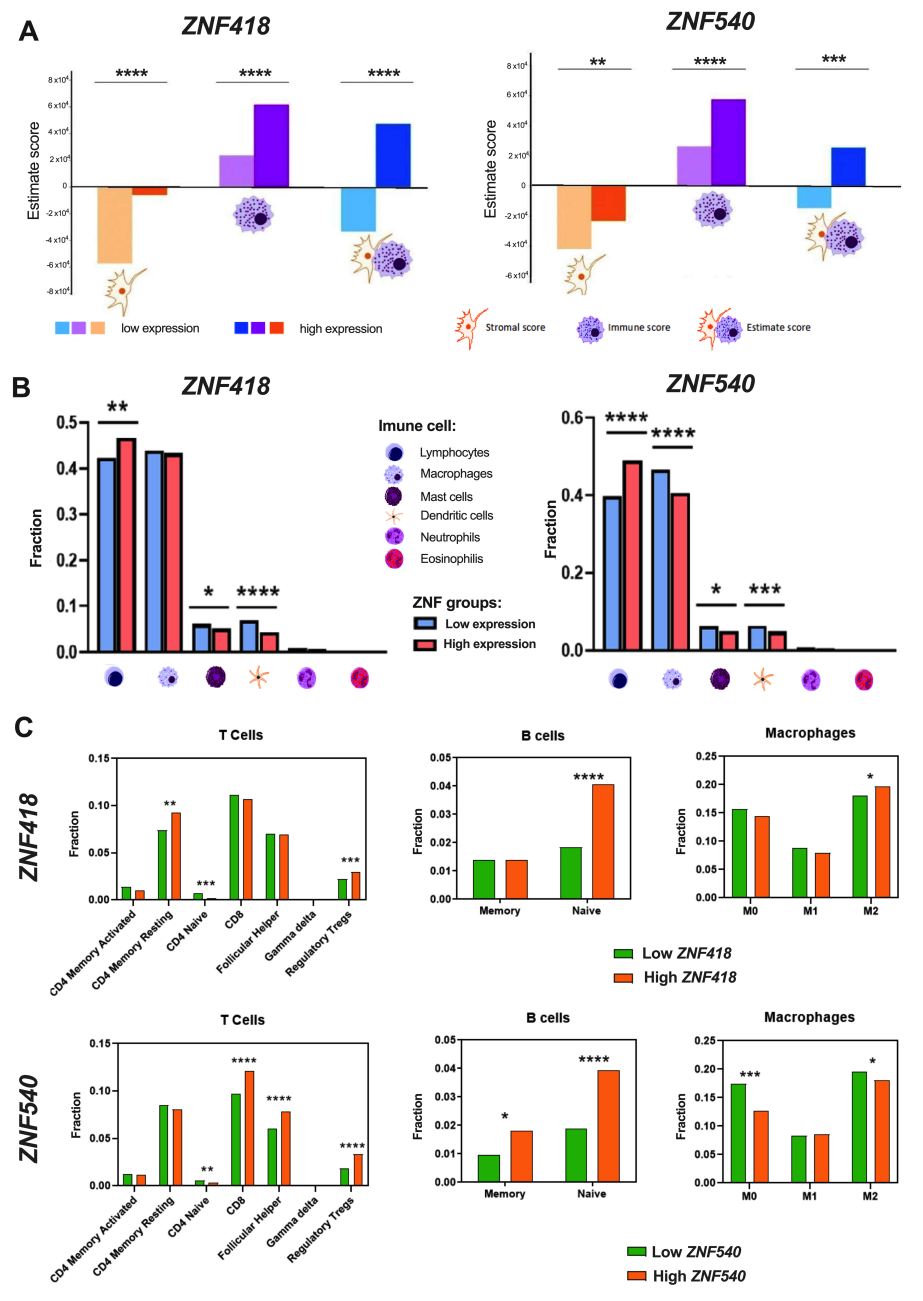
The immunological profile of HNSCC patients depends on the low and high levels of *ZNF418* (*n_Low_* = 271, *n_High_* = 250) and *ZNF540* (*n_Low_* = 261, *n_High_* = 260) genes. (**A**) Stromal, Immune, and ESTIMATE scores; (**B**) Infiltration of specific immune cells in tumor samples; (**C**) Differences in the fraction of T cells, B cells, and macrophages; *t*-test or Mann–Whitney U test; *n*—number of cases; *ns*—not significant; * *p* ≤ 0.05; ** *p* ≤ 0.01; *** *p* ≤ 0.001; **** *p* ≤ 0.0001 are considered significant.

We further investigated the immunological profile of HNSCC tumors, depending on the low and high levels of *ZNF418* and *ZNF540* genes. First, the score of stromal cells, immune cells, and finally the ESTIMATE score were evaluated using the ESTIMATE analysis. An elevated fraction of stromal and immune cells were found in the samples with an upregulated expression of *ZNF418* and *ZNF540* (Figure 5A). These factors clearly showed that the ESTIMATE scores of the HNSCC samples differ significantly (*p* < 0.05) depending on ZNF expression (except for *ZNF426*) (Figure 5A and Supplementary Figure S3A).

It was indicated that patients with a higher expression of *ZNF418* and *ZNF540* genes displayed similar immunological profiles, which were manifested by significantly higher levels of lymphocytes and lower levels of mast cells and dendritic cells. Only for *ZNF540* were differences in the fraction of macrophages observed, and patients with higher levels of *ZNF540* displayed lower levels of infiltration of these cells in the tumor mass (Figure 5B). Eosinophils and neutrophils did not show any significant changes (*p* > 0.05) depending on the expression of these two ZNFs (Figure 5B).

Further analysis of specific subpopulations of immune cells indicated that patients with higher levels of *ZNF418* had a higher fraction of CD4+ memory resisting and regulatory Treg cells, and a lower fraction of CD4 naïve T cells. Moreover, higher levels of CD8+, follicular helper cells, and regulatory Treg cells, as well as lower levels of CD4 naïve T cells for patients with higher levels of *ZNF540*, were observed (Figure 5C). In the case of B cells, for patients with higher levels of *ZNF418*, only significantly higher levels of naïve B cells were observed. In contrast to that, higher levels of both naïve and memory B cell subpopulations were indicated in patients with higher levels of *ZNF540*(Figure 5C). The last analyzed subtypes were macrophages. In the case of higher expression levels of *ZNF418*, a significantly increased population of M2 macrophages was observed. In contrast to that, the reduced (*p* < 0.05) fraction of M0 and M2 populations was characteristic for the tumors with higher expressions of *ZNF540* (Figure 5C).

As mentioned above, for *ZFP28*, *ZNF132*, *ZNF426*, and *ZNF880*, no associations with DFI and OS were observed; however, immunological profiling depending on the expression levels of these ZNFs was carried out. Patients with higher levels of *ZFP28*, *ZNF132*, and *ZNF880* displayed similar immune profiles with a higher fraction of lymphocytes and lower levels of macrophages and dendritic cells. Only for *ZNF426* were significant differences (*p* < 0.05) indicated for mast cells, dendritic cells, as well as neutrophils (Supplementary Figure S3C). Moreover, the analysis of specific subpopulations of T cells showed that patients with higher levels of ZNFs displayed higher levels of resistant CD4+ memory cells. It should be noted that changes in the expression levels of *ZNF426* were associated with differences in most subtypes of T cells. A higher fraction of naïve B cells was observed for *ZFP28*, *ZNF132*, and *ZNF880*, and only patients with higher levels of *ZNF426* displayed a lower fraction of memory B cells. Surprisingly, macrophage profiles differed the most in the analyzed immunological cells depending on ZNF levels, and significant differences were observed only in the case of M1 and M0 subtypes for *ZFP28* and *ZNF132*, respectively. All results are presented in Supplementary Figure S3C.

### 3.5. Validation of ZNF540 as a Potential Biomarker Using GEO Data

Next, we utilized the GSE65858 dataset to validate the possible association of *ZNF540* expression with *HPV* status that was identified in our previous analysis based on the TCGA data.

**Figure 6:**
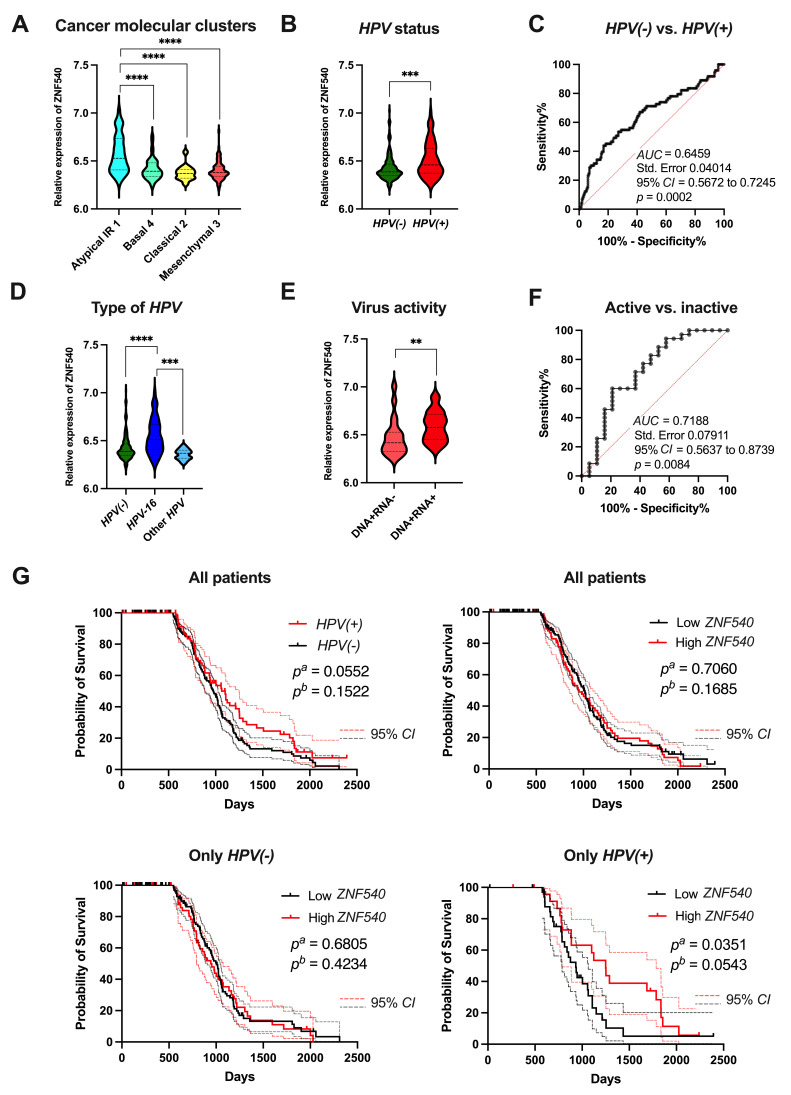
Validation of *ZNF540* in HNSCC patients using GSE65858 dataset: (**A**) *ZNF540* level in different types of HNSCC divided into cancer molecular clusters (atypical IR1, basal 4, classical 2, mesenchymal 3; *n* = 73 vs. 84 vs. 30 vs. 83, respectively); (**B**) expression level of *ZNF540* depending on *HPV* status; and (**C**) Receiver operating characteristic (ROC) analysis of ZNF540’s ability to distinguish groups *HPV(+)* vs. *HPV(−)* (*n* = 94 vs. 176); (**D**) expression level depending on the type of *HPV (HPV(−)* vs. *HPV-16* vs. other types of *HPV*, *n* = 196 vs. 61 vs. 13); (**E**) association between *ZNF540* and virus activity [*HPV(+)* (DNA+/RNA+) vs. *HPV(+)* (DNA+/RNA−), *n* = 35 vs. 19] with (**F**) ROC analysis; (**G**) the overall survival (OS) of HNSCC patients depending on the *HPV* status (*n* = 196 vs. 73) and *ZNF540* expression levels in all cases (*n_Low_* = 176, *n_High_* = 94), *HPV(−)* (*n_Low_* = 123, *n_High_* = 72) and *HPV(+)* (*n_Low_* = 35, *n_High_* = 25) patients with 95% CI marked as lighter lines. The graphs show median values; Mann–Whitney U test or one-way ANOVA test with post-test; *n*—number of cases, CI—confidence interval; *ns*—not significant, ** *p* < 0.01, *** *p* < 0.001, **** *p* < 0.0001; *p^a^*—log-rank (Mantel–Cox) test, *p^b^*—Gehan–Breslow–Wilcoxon Test; *p* < 0.05 considered as significant.

The expression level of *ZNF540* was also assessed in four different molecular cancer clusters. The highest expression of *ZNF540* was observed in the “atypical (IR) 1” cluster compared to the “basal 4”, “classical 2”, and “mesenchymal 3” types of HNSCCs (Figure 6A). Moreover, we tested the association between the levels of *ZNF540* expression and various clinicopathological parameters and we found some significant differences only in the case of smoking. Interestingly, no differences in ZNF540 expression were observed between various tumor localizations (*p* > 0.05). All data are presented in Supplementary Table S4.

First of all, our results from the GSE65858 dataset confirmed the upregulation of *ZNF540* expression in the case of *HPV(+)* compared to *HPV(−)* HNSCC samples (6.51 ± 0.021 vs. 6.43 ± 0.009; *p* = 0.0002) (Figure 6B). The ROC analysis also indicated the high ability of *ZNF540* to distinguish *HPV*-positive and negative patients (AUC = 0.65; *p* = 0.0002) (Figure 6C). Moreover, we observed that the patients infected with *HPV* type 16 had a higher expression of *ZNF540* in comparison to other types of *HPV* (6.55 ± 0.023 vs. 6.36 ± 0.015; *p* = 0.0001) (Figure 6D). Next, the expression levels of *ZNF540* were examined depending on the virus activity. Its upregulation was demonstrated in the group of *HPV(+)* (DNA+/RNA+) vs. *HPV(+)* (DNA+/RNA−) (6.59 ± 0.027 vs. 6.48 ± 0.046; *p* = 0.008). The ROC analysis and estimation of the AUC revealed that the *ZNF540* expression level may be utilized to discriminate between an active and inactive *HPV* infection status with high specificity and sensitivity (AUC = 0.72; 95% CI = 0.56 to 0.87; *p* = 0.0084) (Figure 6E,F).

Patients from the GSE65858 dataset were divided based on the *HPV* status, and a between-group OS was calculated. The results suggest a slightly better outcome of the *HPV(+)* patients compared to the *HPV(−)* patients (*p* = 0.0552). For the verification of the possible usage of *ZNF540* expression level as a prognostic marker, we divided HNSCC patients into the following subgroups: all patients (*HPV(+)* and *HPV(−)*), only *HPV(−)*, and only *HPV(+)*. Each of the subgroups was further divided into high and low expression groups based on the mean *ZNF540* expression. For all patients and for *HPV(−)* patients, no differences in overall survival were observed between groups with a low and high expression of *ZNF540* (*p* = 0.7060 and *p* = 0.6805, respectively). However, a significantly better OS was observed for the *HPV(+)* patients with a higher level of *ZNF540* compared to the low expression group, with a median of survival of 1249 vs. 933 days, respectively, (95% CI = 0.3093 to 0.9909; *p* = 0.0351) (Figure 6G).

## 4. Discussion

The zinc finger proteins (ZNFs) are one of the most abundant proteins encoded in the human genome. However, due to the vast complexity of this large family of transcriptional factors, the exact roles of ZNFs are still unexplored. In this study, we analyzed *ZFP28*, *ZNF132*, *ZNF418*, *ZNF426*, *ZNF540*, and *ZNF880* in HNSCC, focusing on their biological role, association with various clinico-pathological parameters, and potential utility as biomarkers. The analysis was carried out using the TCGA data, followed by the validation with an alternative dataset from GEO.

First of all, our results based on the single-gene approach show that the expression of all analyzed ZNFs was lower in HNSCC samples compared to healthy controls, which is a favorable feature for diagnostic biomarkers. This approach confirmed the outcomes of our previous pan-cancer transcriptomic analysis utilizing the TCGA data [21]. Surprisingly, we found that the cross-correlation between the expression of ZNFs was only marginal. Of note, our ROC curve test showed a very good capacity for each ZNF to discriminate between tumor and normal samples, further pinpointing their potential applicability as biomarkers.

Although none of the existing studies explored the above transcripts in more detail in HNSCC, our data align with other published observations. For example, *ZNF132* was shown to be epigenetically silenced via promoter hypermethylation in HNSCC [23,24], esophageal squamous cell carcinoma (ESCC) [20], and lung adenocarcinoma (LUAD) [29]. Phenotypically, the cells with reduced *ZNF132* expression had decreased mobility in LUAD [29] and growth, migration, invasion, and tumorigenicity in ESCC [20]. These observations suggest the tumor suppressor function of *ZNF132*. High promoter methylation was also reported in the case of *ZNF418* in laryngeal squamous cell carcinoma [25] and *ZNF540* in clear cell renal cell carcinoma [18]. Moreover, the study by Hui et al. indicated that *ZNF418* was significantly downregulated in gastric carcinoma patients [19]. In contrast, *ZNF880* and *ZFP28* were found upregulated in colorectal cancer [16] and melanoma [15], respectively. Interestingly, promoter hypermethylation frequently leads to the epigenetic inactivation of *ZNF* genes with the TSG features in various cancer types [14]. Thus, it is likely that at least some of the above-analyzed ZNFs may also become downregulated via the CpG methylation mechanism in HNSCC, and this possibility warrants further studies.

We further investigated whether the mRNA expression of selected ZNFs may differ depending on various clinico-pathological parameters. We observed particular similarities between *ZFP28*, *ZNF540*, and *ZNF132* signatures. First, we found that the expression level of most ZNFs (apart from *ZNF880*) depended on tumor location. The pharynx was the site of the highest expression for *ZFP28*, *ZNF540*, and *ZNF132*, and the lowest for *ZNF426*. Of note, *ZNF132* and *ZNF540* expression differed in all three anatomical sites: oral cavity, larynx, and pharynx. Secondly, *ZFP28*, *ZNF540*, and *ZNF132* were downregulated in tumors with a higher T stage, in older patients, and in the cohort that underwent lymph node dissection from the neck. In contrast, these factors demonstrated an increased expression in high-grade tumors (G3 + G4). For further explanation of whether these ZNFs were associated with more aggressive forms of HNSCC, we analyzed their expression profile in different molecular subtypes. We observed that all examined ZNFs were upregulated in the mesenchymal and less aggressive, atypical [30] tumors.

According to our knowledge, there are no comprehensive studies on the expression of ZNFs in the context of HNSCC and related risk factors. Here, we demonstrate that a higher *ZFP28* expression was associated with alcohol consumption, whereas smoking was related to higher *ZFP28*, *ZNF880*, and *ZNF418* levels and lower *ZNF132* expression. Moreover, we observed that *ZNF132* and *ZNF540* were upregulated, and *ZNF426* was downregulated in the *HPV(+)* group compared to the *HPV(−)* group. Although *ZNF132* expression and promoter methylation were analyzed previously in *HPV(+)* HNSCC cases, no association with *HPV* was demonstrated [24]. Such a discrepancy between our study and [24] may reflect ethnic differences between populations analyzed or may be due to the lower number of patients included in [24].

Since their biological roles remain largely uncharacterized, we further sought to determine ZNFs’ involvement in tumor-associated pathways. Using REACTOME and GSEA tools, we confirmed the correlation of ZNFs with various signaling pathways engaged in tumorigenesis and immune responses. In summary, those pathways include MAPK, NF-κB, TNF, JNK, and RAS signaling. For example, *ZNF418* and *ZNF540* expression was linked to KRAS signaling. In addition, *ZNF540* expression positively correlated with defective base excision repair associated with OGG1 and with the expression of the TNF receptor superfamily (TNFSF) that mediates non-canonical NF-κB signaling, which is essential for immune response and cell growth regulation [31]. Our data also indicate that both *ZNF540* and *ZNF418* are associated with *IL-2* signaling (and *IL-15* and *IL-21* in the case of *ZNF418*). Moreover, we found that both factors were related to altered immunological profiles in HNSCC patients. Thus, it may be hypothesized that the decreased expression of *ZNF540* and *ZNF418* may affect tumor formation not only through various oncogene-related pathways but also via interfering with the immune response.

Furthermore, our study, for the first time, demonstrates that *ZNF418* and *ZNF540* expressions could be used as potential biomarkers in HNSCC. Notably, the patients with increased levels of *ZNF418* showed significantly longer OS, while those with higher *ZNF540* expression had prolonged disease-free intervals. Other reports may indirectly support our findings. For example, high *ZNF418* expression correlated with improved OS in gastric carcinoma [18], whereas its promoter hypermethylation showed a good discriminatory potential between high- and low-risk patient cohorts [25]. Additionally, Arai et al. determined that *ZNF540* is frequently methylated in clear renal cell carcinoma patients with worse survival [18]. Of note, our data reveal a link between *ZNF540* expression and NF-κB, MAPK, and JNK pathways, which contribute to the epithelial-to-mesenchymal transition (EMT) [32,33]. Hypothetically, *ZNF540* may interfere with the EMT events, thus lowering the chance of local and distant metastases and improving the prognosis of HNSCC patients. Nevertheless, these hypotheses require further clinical and wet-lab investigation.

Importantly, our detailed analysis of the potential clinical usage of ZNFs revealed that *ZNF540* expression might serve as a prognostic marker in the context of *HPV* infection. Based on the TCGA and GEO data, we showed that the *ZNF540* level is higher in *HPV(+)* patients than in *HPV(−)* patients. Moreover, we indicated that a higher expression of *ZNF540* is observed in patients with an active *HPV* infection. So far, only one study revealed that *ZNF540* is upregulated in *HPV(+)* active vs. *HPV(+)* inactive patients, as well as *HPV(+)* active and *HPV(+)* inactive in comparison to *HPV(−)* HNSCC patients [34]. However, no in vitro studies describe the biological role of *ZNF540*. As mentioned previously, the highest *ZNF540* expression was observed in the HNSCC molecular subtype characterized as “atypical”. It was indicated that the atypical subtype was a less aggressive type of HNSCC and was associated with a strong immune signature [35]. Based on our immunological profile results, we observed that patients with higher levels of ZNF540 displayed higher levels of CD8, follicular helper T cells, memory and naive B cells, and lower levels of M2 macrophages. Cillo et al. described the immune landscape of viral- and carcinogen-driven HNSCC and indicated that a higher level of follicular helper T cells was associated with longer progression-free survival [35]. We also observed in the TCGA data that patients with higher levels of *ZNF540* displayed longer disease-free intervals. While the TCGA dataset comprised all patients, *HPV(+)* and *HPV(−)*, further analysis based on the GEO dataset clearly showed that the *HPV(+)* patients with higher levels of *ZNF540* had significantly longer overall survival.

In conclusion, we showed that *ZFP28*, *ZNF132*, *ZNF418*, *ZNF426*, *ZNF540*, and *ZNF880* had reduced expression in HNSCC compared to healthy tissues. Moreover, their expression levels were associated with various clinical parameters, risk factors, and signaling pathways crucial for tumorigenesis and immune responses. We revealed that the high expression of *ZFP540* and *ZFP418* correlated with a favorable prognosis in HNSCC. Specifically, high *ZFP540* levels were associated with improved survival of *HPV(+)* patients. Altogether, our findings emphasize the potential applicability of *ZNF418* and *ZNF540* as prognostic biomarkers in HNSCC. These promising data open new avenues for additional research to dissect the mechanisms responsible for ZNF downregulation. The limitation of our study is that it is based on the TCGA and GEO data, where we had no control over the quality of samples and their sequencing. However, in both data sets, different methodologies were implicated, and the results are similar, which confirms that *ZNF540* is closely associated with *HPV* infection. More importantly, however, the molecular mechanisms contributing to the *ZNF540* involvement in HNSCC biology are unknown and need to be clarified in the in vitro cell line models and in vivo based on large patient samples with known *HPV* status.

## Figures and Tables

**Table 1:** Expression levels of *ZFP28*, *ZNF132*, *ZNF418*, *ZNF426*, *ZNF540*, and *ZNF880* depending on clinical-pathological parameters. *t*-test or Mann–Whitney U test was used to assess the statistical significance; *n*—number of cases. The differences with *p* < 0.05 were considered as significant and marked in bold in the specified cell.

		*FP28*			*ZNF880*			*ZNF540*			*ZNF418*			*ZNF426*			*ZNF132*		
Parameter	Group	Mean ± SEM	*p*-Val	*n*	Mean ± SEM	*p*-Val	*n*	Mean ± SEM	*p*-Val	*n*	Mean ± SEM	*p*-Val	*n*	Mean ± SEM	*p*-Val	*n*	Mean ± SEM	*p*-Val	*n*
Age	<61	−1.666 ± 0.1029	**0.0284**	258	−1.753 ± 0.08617	0.4497	258	−2.023 ± 0.07620	**0.0016**	258	−2.436 ± 0.07546	0.0429	258	0.2168 ± 0.09069	0.9285	258	−1.024 ± 0.05959	**0.0020**	258
	>61	−1.971 ± 0.09452		263	−1.848 ± 0.09072		263	−2.354 ± 0.07172		263	−2.736 ± 0.07637		263	0.2706 ± 0.07680		263	−1.273 ± 0.05513		263
Gender	Female	−1.878 ± 0.1224	0.6950	137	−1.856 ± 0.1226	0.5837	137	−2.430 ± 0.09578	**0.0060**	137	−2.655 ± 0.1031	0.2337	137	0.5759 ± 0.10140	**0.0080**	137	−1.328 ± 0.06942	**0.0043**	173
	Male	−1.796 ± 0.08431		385	−1.778 ± 0.07269		385	−2.102 ± 0.06225		385	−2.513 ± 0.06598		385	0.1259 ± 0.07089		385	−1.087 ± 0.04916		385
Alcohol	Positive	−1.726 ± 0.08599	**0.0480**	348	−1.752 ± 0.07466	0.1381	348	−2.151 ± 0.06220	0.2524	348	−2.516 ± 0.06876	0.2158	348	0.2608 ± 0.07361	0.1041	348	−1.094 ± 0.04794	0.0568	348
	Negative	−2.027 ± 0.1232		163	−1.952 ± 0.1160		163	−2.283 ± 0.1014		163	−2.683 ± 0.09407		163	0.2041 ± 0.1056		163	−1.272 ± 0.07984		163
Smoking	No/Ex	−1.994 ± 0.08627	**0.0004**	333	−1.902 ± 0.07998	**0.0456**	333	−2.234 ± 0.06583	0.3147	333	−2.664 ± 0.06435	**0.0091**	333	0.2411 ± 0.07058	0.1674	333	−1.232 ± 0.04994	**0.0063**	333
	Yes	−1.508 ± 0.1173		177	−1.638 ± 0.1004		177	−2.122 ± 0.08948		117	−2.332 ± 0.1080		117	0.2861 ± 0.1105		177	−0.9967 ± 0.06593		177
Cancer Stage	I + II	−1.744 ± 0.1480	0.1890	101	−1.771 ± 0.1227	0.6981	101	−2.212 ± 0.1121	0.5988	101	−2.579 ± 0.1212	0.8857	101	0.4043 ± 0.12760	0.3499	101	−1.167 ± 0.06949	0.8409	101
	III + IV	−1.961 ± 0.08416		349	−1.832 ± 0.07689		349	−2.282 ± 0.06302		349	−2.573 ± 0.06828		349	0.2019 ± 0.07327		349	−1.211 ± 0.04962		349
T stage	T1 + T2	−1.719 ± 0.1111	**0.0325**	185	−1.736 ± 0.09599	0.3174	185	−2.005 ± 0.08372	**<0.0001**	185	−2.470 ± 0.08898	0.2256	185	0.2275 ± 0.09747	0.5552	185	−1.057 ± 0.06083	**0.0317**	185
	T3 + T4	−2.029 ± 0.09507		274	−1.869 ± 0.08743		274	−2.434 ± 0.06896		274	−2.640 ± 0.07954		274	0.2760 ± 0.08243		274	−1.266 ± 0.05489		274
N stage	N0 + N1	−1.907 ± 0.1009	0.8130	243	−1.789 ± 0.09103	0.7360	243	−2.241 ± 0.06699	0.9973	243	−2.598 ± 0.07829	0.2244	243	0.4471 ± 0.08127	**0.0012**	243	−1.275 ± 0.05776	**0.0064**	243
	N2 + N3	−1.907 ± 0.1135		179	−1.835 ± 0.1021		179	−2.241 ± 0.09822		179	−2.442 ± 0.1032		179	−0.03371 ± 0.1085		179	−1.042 ± 0.06325		179
Grade	G1 + G2	−1.968 ± 0.08064	**0.0033**	368	−1.871 ± 0.07577	0.1031	368	−2.342 ± 0.06235	**<0.0001**	368	−2.692 ± 0.06427	**0.0001**	368	0.3830 ± 0.06992	**0.0001**	368	−1.262 ± 0.04763	**0.0012**	368
	G3 + G4	−1.496 ± 0.1444		132	−1.636 ± 0.1149		132	−1.858 ± 0.09752		132	−2.189 ± 0.1152		132	−0.07011 ± 0.1165		132	−0.9401 ± 0.08017		132
Perineural Invasion	Positive	−2.005 ± 0.1118	0.4205	169	−1.857 ± 0.1085	0.3722	169	−2.493 ± 0.09021	**0.0188**	169	−2.655 ± 0.09371	0.9498	169	0.3002 ± 0.1025	0.2816	169	−1.307 ± 0.05987	0.0696	169
	Negative	−1.796 ± 0.1205		195	−1.727 ± 0.09790		195	−2.138 ± 0.08013		195	−2.575 ± 0.09091		195	0.1339 ± 0.1063		195	−1.125 ± 0.06886		195
Lymph Node Neck Dissection	Positive	−1.915 ± 0.07603	**0.0044**	422	−1.800 ± 0.06881	0.8567	422	−2.255 ± 0.05758	**0.0085**	422	−2.555 ± 0.06305	0.8512	422	0.2269 ± 0.06788	0.6087	422	−1.207 ± 0.04532	**0.0079**	422
	Negative	−1.370 ± 0.1726		97	−1.771 ± 0.1533		97	−1.892 ± 0.1286		97	−2.519 ± 0.1193		97	0.2672 ± 0.11610		97	−0.8949 ± 0.09270		97
Angio- lymphatic Invasion	Positive	−1.765 ± 0.1361	0.2468	125	−1.694 ± 0.1156	0.3401	125	−2.139 ± 0.1134	**0.0426**	125	−2.484 ± 0.1129	0.0888	125	−0.01257 ± 0.1324	**0.0161**	125	−1.082 ± 0.07789	0.0709	125
	Negative	−1.962 ± 0.1060		225	−1.842 ± 0.09593		225	−2.397 ± 0.07049		225	−2.716 ± 0.07975		225	0.3853 ± 0.09004		225	−1.283 ± 0.05889		225
*HPV p16* status	Positive	−1.060 ± 0.3101	0.0785	39	−1.761 ± 0.2281	0.2259	39	−0.9781 ± 0.1946	**<0.0001**	39	−2.332 ± 0.2203	0.8762	39	−0.5185 ± 0.2457	**0.0002**	39	−0.3564 ± 0.1596	**<0.0001**	39
	Negative	−1.544 ± 0.1639		73	−1.416 ± 0.1680		73	−2.173 ± 0.1262		73	−2.374 ± 0.1541		73	0.4726 ± 0.1646		73	−1.072 ± 0.09748		73

## Data Availability

The datasets used and/or analyzed during the current study are available from the corresponding author on reasonable request. Raw data are available in the TCGA and GEO databases.

## Supplementary Materials

The following supporting information can be downloaded at: https://www.mdpi.com/article/10.3390/curroncol29120779/s1, Figure S1. Heat map and clustering of mean expression levels of ZNFs depending on the specific clinical parameters; Figure S2. (A) Disease-free interval (DFI) and (B) overall survival (OS) of HNSCC patients depending on the ZFP28, ZNF880, ZNF426, and ZNF132 expression levels, respectively. Results presented for 5 years of observation, low and high subgroups of patients divided on the mean of expression; *p^a^*—Long-rank (Mantel-Cox) test; *p^b^*—Gehan-Breslow-Wilcoxon test; *p* < 0.05 considered significant; Figure S3. The immunological profile of HNSCC patients depends on the low and high levels of ZFP28, ZNF132, ZNF426, and ZNF880 transcripts. (A) Stromal, immune, and ESTIMATE scores; (B) Infiltration of specific immune cells in tumor samples; (C) Differences in the fraction of T cells, B cells, and macrophages; ns—not significant; * *p* ≤ 0.05; ** *p* ≤ 0.01; *** *p* ≤ 0.001; **** *p* ≤ 0.0001 considered as significant; Table S1. Number of patients’ cases analyzed in the groups depending on the specific clinical parameters; *n*—number of cases; Table S2. Collected genes assigned to pathways positively and negatively correlated with ZFP28, ZNF880, ZNF540, ZNF418, ZNF426, and ZNF132; Table S3. Involvement of ZNFs transcripts in cellular processes based on GSEA analysis in HNSCC patients. Normalized enrichment scores for GSEA analysis of MSigDB gene sets for oncogenic and hallmark gene sets in the group of patients with low and high expression levels of specified ZNFs. Only results set with *p* ≤ 0.05 and FDR ≤ 0.25 were shown. NES (normalized enrichment score), *p-val* (nominal *p*-value), and FDR *q-val* (false discovery rate); Table S4. Expression level of ZNF540 depending on clinical-pathological parameters in HNSCC patients from GSE65858; Mann-Whitney U test or one-way ANOVA, *p* < 0.05 considered as significant.
