# Supplementary material for: Zinc Finger Proteins in Head and Neck Squamous Cell Carcinomas: ZNF540 May Serve as a Biomarker
Source: Curr Oncol. 2022 Dec 16;29(12):9896–915. doi: 10.3390/curroncol29120779 (PMC9776630; doi:10.3390/curroncol29120779)
Supplement: Supplementary file 1 [file curroncol-29-00779-s001.zip › curroncol-2039063-supplementary.pdf]

Current Oncology

Supplementals

Zinc Finger Proteins in Head and Neck Squamous Cell Carcinomas: ZNF540 May Serve as a Biomarker

Joanna Sobocińska [1,2,3], Joanna Nowakowska [3,6], Sara Molenda [3], Anna Olechnowicz [3,7], Kacper Guglas [1,2,4,*], Joanna Kozłowska-Masłoń [1,2,5,*], Urszula Kazimierczak [3,†], Marta Machnik [3,†], Urszula Oleksiewicz [3,†], Anna Teresiak [1,2], Katarzyna Lamperska [1,2] and Tomasz Kolenda [1,2]

Table S1. Number of patients' cases analyzed in the groups depending on the specific clinical parameters; *n* - number of cases.

ZNF		*ZFP28*	*ZNF880*	*ZNF540*	*ZNF418*	*ZNF426*	*ZNF132*
**Parameter**	**Group**	**n**	**n**	**n**	**n**	**n**	**n**
Age	< 61	258	258	258	258	258	258
	> 61	263	263	263	263	263	263
Gender	Female	137	137	137	137	137	173
	Male	385	385	385	385	385	385
Alcohol	Positive	348	348	348	348	348	348
	Negative	163	163	163	163	163	163
Smoking	No/Ex	333	333	333	333	333	333
	Yes	177	177	117	117	177	177
Cancer Stage	I + II	101	101	101	101	101	101
	III + IV	349	349	349	349	349	349
T stage	T1 + T2	185	185	185	185	185	185
	T3 + T4	274	274	274	274	274	274
N stage	N0 + N1	243	243	243	243	243	243
	N2 + N3	179	179	179	179	179	179
Grade	G1 + G2	368	368	368	368	368	368
	G3 + G4	132	132	132	132	132	132
Perineural Invasion	Positive	169	169	169	169	169	169
	Negative	195	195	195	195	195	195
Lymph Node Neck Dissection	Positive	422	422	422	422	422	422
	Negative	97	97	97	97	97	97
Angiolymphatic Invasion	Positive	125	125	125	125	125	125
	Negative	225	225	225	225	225	225
HPV p16 status	Positive	39	39	39	39	39	39
	Negative	73	73	73	73	73	73

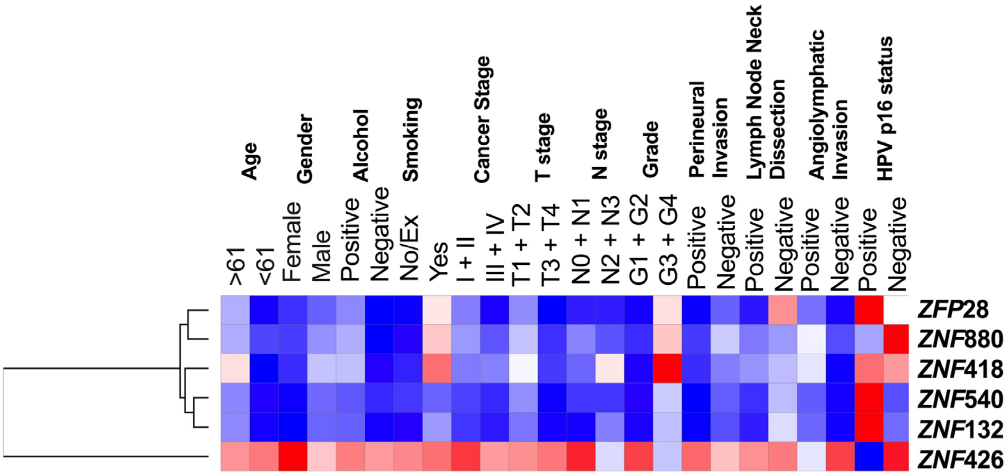

Figure S1. Heat map and clustering of mean expression levels of ZNFs depending on the specific clinical parameters.

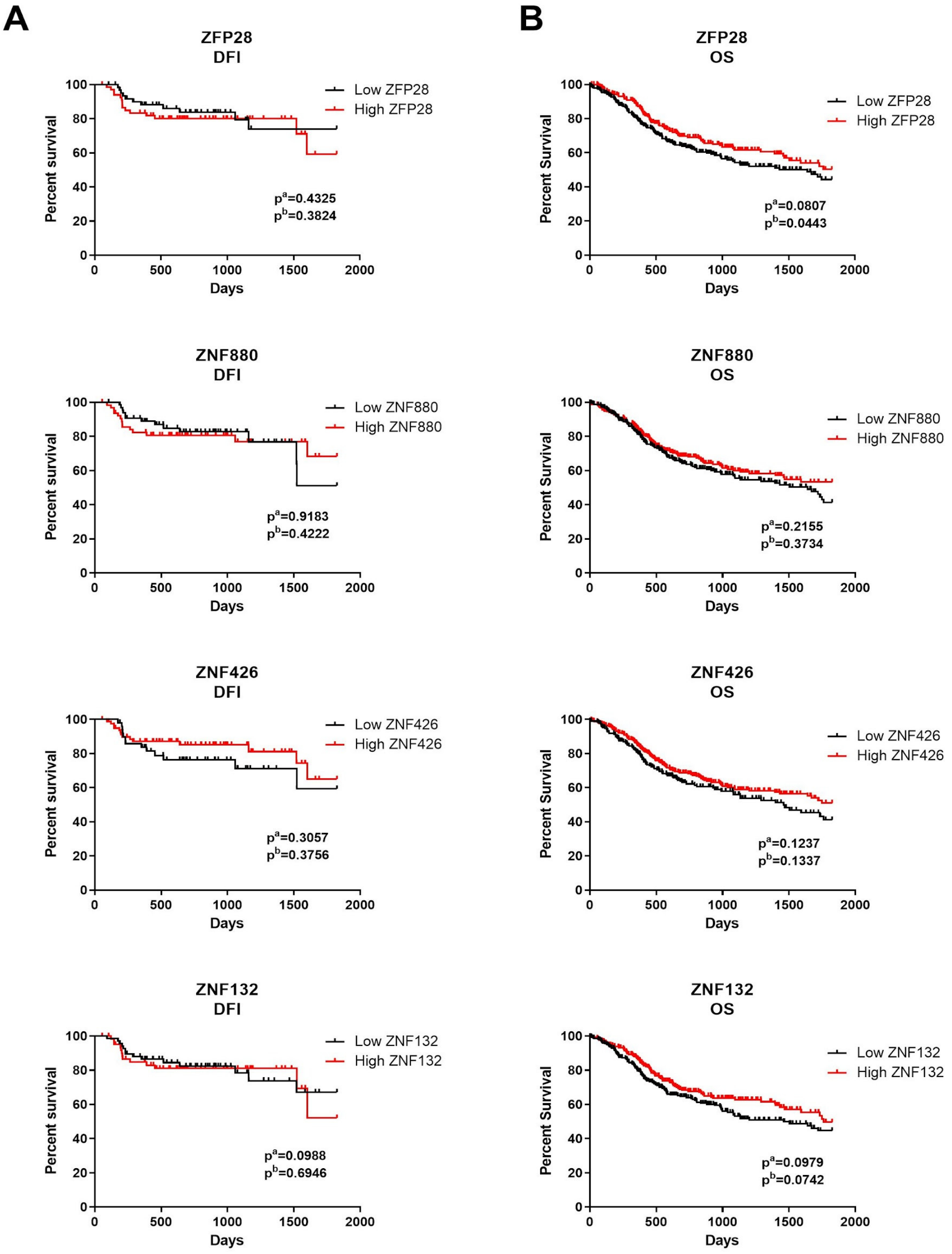

Figure S2. A) Disease-free interval (*DFI*) and B) overall survival (*OS*) of HNSCC patients depending on the *ZFP28, ZNF880, ZNF426* and *ZNF132* expression level, respectively. Results presented for 5 years observation, low and high subgroups of patients divided on the mean of expression; p^a - Long-rank (Mantel-Cox) test; p^b - Gehan-Breslow-Wilcoxon test; $p < 0.05$ considered significant.

Table S2. Collected genes assigned to pathways positively and negatively correlated with *ZFP28, ZNF880, ZNF540, ZNF418, ZNF426*, and *ZNF132*.

**Positively correlated genes with ZFP28**	
**Defective CHST6 causes MCDC1**	*PRELP, KERA, LUM, OMD, OGN, CHST6, FMOD*

Voltage gated Potassium channels	*KCNQ3, KCNQ1, KCNH2, KCND1, KCNG3, KCNH4, KCNK2, KCNA3, KCNA6, KCNAB2, KCNA5, KCNA2, KCNC3, KCNB2, CNKSR2*
Molecules associated with elastic fibers	*FBLN1, LTBP3, ELN, EFEMP2, ITGB3, MFAP4, FBLN5, EMILIN1, FBLN2, GDF5, FBN1, ITGA8*
Defective B4GALT1 causes B4GALT1-CDG (CDG-2d)	*PRELP, KERA, LUM, OMD, OGN, FMOD*
Defective ST3GAL3 causes MCT12 and EIEE15	*PRELP, KERA, LUM, OMD, OGN, FMOD*
Elastic fiber formation	*FBLN1, EFEMP2, ITGB3, EMILIN1, FBN1, ITGA8, LOXL3, LOXL4, LTBP3, ELN, DRP2, MFAP4, FBLN5, FBLN2, LOXL1, GDF5*
Collagen chain trimerization	*COL9A2, COL6A6, COL8A1, COL6A3, COL8A2, COL9A1, COL4A3, COL14A1, COL4A4, COL15A1, COL19A1*
Diseases associated with glycosaminoglycan metabolism	*PRELP, CHST14, GPC2, GPC3, OGN, SDC2, CHST6, VCAN, FMOD, KERA, LUM, OMD, PAPSS2, HSPG2, DCN*
Nuclear Receptor transcription pathway	*NR2F1, RARB, RORB, PGR, NR1D2, THRA, NR1I2, AR, NR5A2, NR2C2, ESRRG, NR3C2*
Post-transcriptional silencing by small RNAs	*AGO1, TNRC6C, TNRC6B*
**Negatively correlated genes with ZFP28**	
Transport of connexons to the plasma membrane	*TUBA4A, TUBB4B, TUBA1C, GJB2, TUBB6*
Prefoldin mediated transfer of substrate to CCT/TriC	*TUBA4A, TUBB4B, TUBA1C, TUBB6, CCT3*
Formation of the cornified envelope	*PKP3, KRT14, KRT16, PKP1, KRT17, JUP, KRT6A, KRT6B KRT6C, KRT5, KLK8*
Microtubule-dependent trafficking of connexons from Golgi to the plasma membrane	*TUBA4A, TUBB4B, TUBA1C, TUBB6*
Neutrophil degranulation	*GSTP1, PKP1, FABP5, GM2A, ALDOA, PYCARD, S100A11, ATP6V1D, HMOX2, PA2G4, PYGL, S100A12, TUBB4B, JUP,*

	ANXA2, TOLLIP, PGAM1, PNP, AGPAT2, DYNLL1
Gap junction trafficking	*TUBA4A, TUBB4B, GJB5, GJB6, TUBA1C, GJB3, CLTB, GJB2, TUBB6*
Gap junction assembly	*TUBA4A, TUBB4B, GJB5, GJB6, TUBA1C, GJB3, GJB2, TUBB6*
Gap junction trafficking and regulation	*TUBA4A, TUBB4B, GJB5, GJB6, TUBA1C, GJB3, CLTB, GJB2, TUBB6*
Carboxyterminal post-translational modifications of tubulin	*TUBA4A, TUBB4B, TUBA1C, TUBB6*
Keratinization	*PKP3, KRT14, KRT16, PKP1, KRT17, JUP, KRT6A, KRT6B, KRT6C, KRT5, KLK8*

**Positively correlated genes with ZNF880**	
**Molecules associated with elastic fibers**	*FBLN1, CD93, EFEMP2, EMILIN1, EMILIN2, FBN1, ITGA8, LTBP3, TGFB3, ELN, MFAP4, FBLN5, GDF5, FBLN2*
Elastic fiber formation	*FBLN1, CD93, EFEMP2, EMILIN1, EMILIN2, FBN1, ITGA8, LOXL3, LTBP3, TGFB3, ELN, MFAP4, FBLN5, GDF5, LOXL1, FBLN2*
Defective CHST6 causes MCDC1	*PRELP, KERA, LUM, OMD, OGN, CHST6, FMOD*
Collagen chain trimerization	*COL6A5, COL6A6, COL8A1, COL6A3, COL8A2, COL6A1, COL4A3, COL14A1, COL4A4, COL3A1, COL1A2, COL15A1, COL19A1*
Defective B3GALTL causes Peters-plus syndrome (PpS)	*THSD7A, ADAMTS5, ADAMTS18, SEMA5A, ADAMTS3, ADAMTS2, ADAMTS9, ADAMTS8, ADAMTS10, ADAMTS12, ADAMTSL1, SPON1, CFP*
O-glycosylation of TSR domain-containing proteins	*THSD7A, ADAMTS5,ADAMTS18, SEMA5A, ADAMTS3, ADAMTS2, ADAMTS9, ADAMTS8, ADAMTS10, ADAMTS12, ADAMTSL1, SPON1, CFP, TMEM170B*
Defective B4GALT1 causes B4GALT1-CDG (CDG-2d)	*PRELP, KERA, LUM, OMD, OGN, FMOD*

Defective ST3GAL3 causes MCT12 and EIEE15	*PRELP, KERA, LUM, OMD, OGN, FMOD*
Diseases associated with glycosaminoglycan metabolism	*PRELP, SDC2, OGN, CHST6, VCAN, FMOD, GPC6, KERA, PSAP, LUM, OMD, PAPSS2, HSPG2, DCN*
Assembly of collagen fibrils and other multimeric structures	*COL6A5, COL6A6, COL8A1, COL6A3, COL8A2, PCOLCE, COL6A1, COL4A3, COL14A1, COL4A4, LOXL3, CTSS, COL3A1, COL1A2, COL15A1, LOXL1*
**Negatively correlated genes with ZNF880**	
Formation of the cornified envelope	*PKP3, KRT17*
Keratinization	*PKP3, KRT17*
RAS GTPase cycle mutants	*HRAS*
Defective factor XII causes hereditary angioedema	*F12*
Defective SERPING1 causes hereditary angioedema	*F12*
Activation of RAS in B cells	*HRAS*
RAS signaling downstream of NF1 loss-of-function variants	*HRAS*
SOS-mediated signalling	*HRAS*
Neutrophil degranulation	*PA2G4, GSTP1, S100A11*

**Positively correlated genes with ZNF540**	
**Terminal pathway of complement**	*C7, C5, C8G, CLU*
Phase 3 - rapid repolarization	*KCNE3, KCNQ1, KCNE2*
Interleukin receptor SHC signaling	*PIK3R3, IL5RA, CSF2RB, INPP5D, IL3RA, JAK3, IL2RG, CSF2RA, IL2*
Defective Base Excision Repair Associated with OGG1	*OGG1*
Voltage gated Potassium channels	*KCNA3, KCNA5, KCNAB2, KCNA2, KCNC3, KCNH8, KCNQ1, CNKSR2, KCND1, KCNH4, KCNAB3*

TNF receptor superfamily (TNFSF) members mediating non-canonical NF-kB pathway	*LTA, PDE4C, LTB, CD40LG, TNFSF12, TNFRSF13C, MAP3K14*
**Negatively correlated genes with ZNF540**	
Insulin-like Growth Factor-2 mRNA Binding Proteins (IGF2BPs/IMPs/VICKZs) bind RNA	*IGF2BP2, ACTB, MYC, CD44*
Formation of the cornified envelope	*PKP3, PKP1, PI3, KRT6A, KRT6B, KRT6C, CDSN, KRT14, CAPN1, KRT16, FURIN, KRT17, JUP, LCE3D, KRT5, KLK8*
Transport of connexons to the plasma membrane	*TUBA4A, TUBB3, TUBA1C, GJB2, TUBB6*
Keratinization	*PKP3, PKP1, PI3, KRT6A, KRT6B, KRT6C, CDSN, KRT14, CAPN1, KRT16, FURIN, KRT17, JUP, LCE3D, KRT5, KLK8*
Prefoldin mediated transfer of substrate to CCT/TriC	*ACTG1, TUBA4A, TUBB3, TUBA1C, TUBB6, ACTB*
Signaling by MAPK mutants	*DUSP6, DUSP7*
RHO GTPases activate IQGAPs	*ACTG1, TUBA4A, TUBB3, TUBA1C, TUBB6, ACTB*
Type I hemidesmosome assembly	*KRT14, ITGB4, KRT5, PLEC, ITGA6*
Gap junction trafficking	*ACTG1, TUBA4A, TUBB3, GJB5, GJB6, GJB3, TUBA1C, GJB2, TUBB6, RAB11FIP5, ACTB*
Microtubule-dependent trafficking of connexons from Golgi to the plasma membrane	*TUBA4A, TUBB3, TUBA1C, TUBB6*

**Positively correlated genes with ZNF418**	
**Defective B3GALTL causes Peters-plus syndrome (PpS)**	*SSPO, THSD7A, THSD7B, ADAMTS5, SEMA5B, ADAMTSL2, ADAMTS18, ADAMTSL3, ADAMTS19, ADAMTS2, ADAMTS9, ADAMTS8, ADAMTS10, ADAMTSL1, ADAMTS16, SBSPON, SPON1, CFP*
O-glycosylation of TSR domain-containing proteins	*SSPO, THSD7A, THSD7B, ADAMTS5, SEMA5B, ADAMTSL2, ADAMTS18, ADAMTSL3, ADAMTS19, ADAMTS2, ADAMTS9, ADAMTS8, ADAMTS10,*

	ADAMTSL1, ADAMTS16, SBSPON, SPON1, FMNL1, TMEM170B, CFP
Voltage gated Potassium channels	*KCNQ4, KCNQ3, KCNQ1, KCND1, KCNK2, KCNA3, KCNA6, KCNAB1, KCNAB2, KCNA5, KCNB1, KCNC1, KCNA2, KCNC3, KCNH8, KCNA1, CNKSR2, KCNAB3*
Defective CHST6 causes MCDC1	*PRELP, KERA, LUM, OGN, OMD, CHST6, FMOD*
Molecules associated with elastic fibers	*FBLN1, CD93, EFEMP2, EMILIN1, FBN1, EMILIN2, ITGA8, LTBP3, TGFB3, ELN, MFAP4, FBLN5, GDF5, FBLN2*
cGMP effects	*ITPR1, PDE2A, PDE1A, PDE1B, PDE5A, KCNMB1, ALDH1A1, PDE9A, GIT2, PDE11A, PDE10A, MRVI1*
Defective B4GALT1 causes B4GALT1-CDG (CDG-2d)	*PRELP, KERA, LUM, OGN, OMD, FMOD*
Defective ST3GAL3 causes MCT12 and EIEE15	*PRELP, KERA, LUM, OGN, OMD, FMOD*
Collagen chain trimerization	*COL6A5, COL6A6, COL6A3, COL8A1, COL8A2, COL14A1, COL4A3, COL4A4, PZP, COL3A1, COL15A1, COL1A2, COL19A1*
Elastic fiber formation	*FBLN1, CD93, EFEMP2, EMILIN1, FBN1, EMILIN2, ITGA8, LOXL3, LOXL4, LTBP3, TGFB3, ELN, DRP2, MFAP4, FBLN5, GDF5, LOXL1, FBLN2*
**Negatively correlated genes with ZNF418**	
Transport of connexons to the plasma membrane	*TUBA4A, TUBB4B, TUBA1C, GJB2, TUBB6*
Formation of the cornified envelope	*KRT14, PKP3, KRT16, PI3, KRT17, JUP, PERP KRT6A, KRT6B*
Gap junction assembly	*TUBA4A, TUBB4B, GJB5, GJB6, TUBA1C, GJB3, GJB2, TUBB6*
Recruitment of NuMA to mitotic centrosomes	*TUBA4A, TUBB4B, TUBA1C, TUBG1, DYNLL1, TUBB6*
Gap junction trafficking	*TUBA4A, TUBB4B, GJB5, GJB6, TUBA1C, GJB3, GJB2, TUBB6*

Gap junction trafficking and regulation	*TUBA4A, TUBB4B, GJB5, GJB6, TUBA1C, GJB3, GJB2, TUBB6*
Microtubule-dependent trafficking of connexons from Golgi to the plasma membrane	*TUBA4A, TUBB4B, TUBA1C, TUBB6*
Keratinization	*KRT14, PKP3, KRT16, PI3, KRT17, JUP, PERP, KRT6A, KRT6B*
Carboxyterminal post-translational modifications of tubulin	*TUBA4A, TUBB4B, TUBA1C, TUBB6,*
Prefoldin mediated transfer of substrate to CCT/TriC	*TUBA4A, TUBB4B, TUBA1C, TUBB6*

**Positively correlated genes with ZNF426**	
**Formation of the cornified envelope**	*SPINK5, KRT6A, TGM1, DSP, KRT6B, KRT6C, TGM5, CDSN, LIPK, SPRR1A, SPRR1B, LIPM, RPTN, LIPN, KLK14, KLK13, KRT78, FLG2, SPRR3, KLK12, LCE3E, DSG1, PKP1, DSG3, SPRR2E, FLG, SPRR2F, SPRR2G, CAMK2D, SPRR2A, SPRR2B, DSC1, IVL, DSC2, SPRR2D, DSC3, PERP, LCE2C, LCE2B, KRT80, PPL, LCE2D*
Keratinization	*SPINK5, KRT6A, TGM1, DSP, KRT6B, KRT6C, TGM5, CDSN, LIPK, SPRR1A, SPRR1B, LIPM, RPTN, LIPN, KLK14, KLK13, KRT78, FLG2, SPRR3, KLK12, LCE3E, DSG1, PKP1, DSG3, SPRR2E, FLG, SPRR2F, SPRR2G, CAMK2D, SPRR2A, SPRR2B, DSC1, IVL, DSC2, SPRR2D, DSC3, PERP, LCE2C, LCE2B, KRT80, PPL, LCE2D*
Antigen processing: Ubiquitination & Proteasome degradation	*UBE4A, RBBP6, HECTD1, UBE2H, BTRC, PRMT9, UFL1, ANAPC1, CDH1, ITCH, LMO7, PJA2, KLHL11, UBA6, CUL5, UBE3C, GAN, AP1G1, RNF217, HERC3, KBTBD7, RNF111, TRIP12, UBR1, UBR2, PSME3, PPP2R5E, UBR4, NEDD4, NPEPPS, HACE1, FBXO11, LNPEP, FBXO30, LTN1*
JNK (c-Jun kinases) phosphorylation and activation mediated by activated human TAK1	*TAB3, NOD2, TAB2, TRAF6, MAP2K4, MAP3K7*

activated TAK1 mediates p38 MAPK activation	*TAB3, NOD2, TAB2, TRAF6, MAP3K7, MAPK14*
Neutrophil degranulation	*ANK3, PGM2, AMPD3, CDK12, ATP11A, ARG1, ATP11B, ACTR2, SEC24A, DSP, DYNC1H1, CEACAM6, NRAS, RAB6A, CDK13, BTRC, GSDMA, RBM12, MAPK1, CAP1, S100A12, QSOX1, ADAM10, RDX, FLG2, RAB10, SERPINB2, HRNR, IGF2R, HPSE, DSG1, PKP1, IQGAP1, CAB39, PTAFR, COPB1, CYFIP1, CXCR2, CD177, DDX3X, SERPINB12, VCL, DSC1, PRSS3, PAFAH1B2, ROCK1, UBR4, MAPK14*
Interleukin-36 pathway	*IL36RN, IL36B, IL1RL2, IL36A, IL1F10*
RAS signaling downstream of NF1 loss-of-function variants	*NRAS, KRAS*
Small interfering RNA (siRNA) biogenesis	*DICER1, AGO4, AGO3*
Signaling by RAF1 mutants	*BRAF, IQGAP1, CAMK2D, NRAS, VCL, KRAS, MAPK1*
**Negatively correlated genes with ZNF426**	
SRP-dependent cotranslational protein targeting to membrane	*RPL35A, RPS2, UBB, RPS19, SSR4, RPS5, SSR2, RPS15, RPS14, TFPT, RPL18, RPS11, RPL39, RPL19, SEC61B, RPL24, RPS20, RPL26, RPL22, RPS9, RPS27L, RPS27, RPL28, RPS24, RPL26L1, RPL13, RPL35, RPL38, RPL10, WBP1, RPL12, RPL34, RPLP1, RPL11, RPL30*
Formation of a pool of free 40S subunits	*RPL35A, RPS2, UBB, RPS19, RPS5, RPS15, RPS14, TFPT, RPL18, RPS11, RPL39, RPL19, RPL24, RPS20, RPL26, RPL22, RPS9, RPS27L, RPS27, RPL28, RPS24, RPL26L1, RPL13, RPL35, RPL38, RPL10, EIF3K, RPL12, RPL34, RPLP1, RPL11, RPL30*
Selenocysteine synthesis	*RPL35A, RPS2, UBB, RPS19, RPS5, EEFSEC, RPS15, RPS14, TFPT, RPL18, RPS11, RPL39, RPL19, RPL24, RPS20, RPL26, RPL22, RPS9, RPS27L, RPS27, RPL28, RPS24, RPL26L1, RPL13, RPL35, RPL38, RPL10, RPL12, RPL34, RPLP1, RPL11, RPL30*

Eukaryotic Translation Termination	*RPL35A, RPS2, UBB, RPS19, RPS5, RPS15, RPS14, TFPT, RPL18, RPS11, RPL39, RPL19, RPL24, RPS20, RPL26, RPL22, RPS9, RPS27L, RPS27, RPL28, RPS24, RPL26L1, RPL13, RPL35, RPL38, RPL10, RPL12, RPL34, RPLP1, RPL11, RPL30*
L13a-mediated translational silencing of Ceruloplasmin expression	*RPL35A, RPS2, UBB, RPS19, RPS5, RPS15, RPS14, TFPT, RPL18, RPS11, RPL39, RPL19, RPL24, RPS20, RPL26, RPL22, RPS9, RPS27L, RPS27, RPL28, RPS24, RPL26L1, RPL13, RPL35, RPL38, RPL10, EIF3K, RPL12, RPL34, RPLP1, RPL11, RPL30*
Nonsense Mediated Decay (NMD) independent of the Exon Junction Complex (EJC)	*RPL35A, RPS2, UBB, RPS19, RPS5, RPS15, RPS14, TFPT, RPL18, RPS11, RPL39, RPL19, RPL24, RPS20, RPL26, RPL22, RPS9, RPS27L, RPS27, RPL28, RPS24, RPL26L1, RPL13, RPL35, RPL38, RPL10, RPL12, RPL34, RPLP1, RPL11, RPL30*
Peptide chain elongation	*RPL35A, RPS2, UBB, RPS19, RPS5, RPS15, RPS14, TFPT, RPL18, RPS11, RPL39, RPL19, RPL24, RPS20, RPL26, RPL22, RPS9, RPS27L, RPS27, RPL28, RPS24, RPL26L1, RPL13, RPL35, RPL38, RPL10, RPL12, RPL34, RPLP1, RPL11, RPL30*
Viral mRNA Translation	*RPL35A, RPS2, UBB, RPS19, RPS5, RPS15, RPS14, TFPT, RPL18, RPS11, RPL39, RPL19, RPL24, RPS20, RPL26, RPL22, RPS9, RPS27L, RPS27, RPL28, RPS24, RPL26L1, RPL13, RPL35, RPL38, RPL10, RPL12, RPL34, RPLP1, RPL11, RPL30*
Mitochondrial translation elongation	*MRPS21, MRPL27, MRPL28, MRPL47, MRPS25, MRPS24, GADD45GIP1, MRPS26, CHCHD1, MRPL9, AIP, MRPL41, USF2, MRPL40, MRPL23, MRPL24, MRPL43, MRPS2, MRPL22, MRPL16, MRPL38, MRPL58, MRPS12, MRPS34, MRPS11, AURKAIP1, ERAL1, MRPL52, MRPL53, MRPL51, MRPL34, MRPL57, MRPL54, MRPL33, MRPL11, MRPL55*

Regulation of expression of SLITs and ROBOs	*RPL35A, RPS2, ELOB, UBB, RPS19, RPS5, RPS15, RPS14, TFPT, RPL18, RPS11, RPL39, RPL19, RPL24, RNPS1, RPS20, RPL26, PSMD4, PSMB3, RPL22, PSMB1, MAGOH, PSMB4, RPS9, PSMB10, RBX1, RPS27L, SEM1, RPS27, RPL28, RPS24, RPL26L1, RPL13, RPL35, RPL38, RPL10, RPL12, RPL34, RPLP1, PSMC1, RPL11, RPL30, PSMC5, PSMA4, MAGOHB*

**Positively correlated genes with ZNF132**	
**Post-transcriptional silencing by small RNAs**	*AGO4, AGO1, TNRC6C, TNRC6B*
Collagen chain trimerization	*COL9A2, COL6A5, COL6A6, COL9A1, COL14A1, COL4A3, COL4A4, DENND4A, COL15A1, COL19A1*
Nuclear Receptor transcription pathway	*NR2F1, RARB, NR1D2, RORB, NR1I2, AR, NR5A2, NR2C2, ESRRG, NR3C2*
Competing endogenous RNAs (ceRNAs) regulate PTEN translation	*AGO4, CNOT6L, AGO1, TNRC6C, TNRC6B*
Voltage gated Potassium channels	*KCNA3, KCNAB1, KCNA5, KCNAB2, KCNA2, KCNB2, KCNC3, CNKSR2, KCND1, KCNH4,*
Molecules associated with elastic fibres	*LTBP3, ELN, MFAP4, SMCHD1, FBLN5, FBLN2, GDF5, ITGA8*
FGFR2 ligand binding and activation	*FGFR2, FGF18, FGF7, FGF2*
XAV939 stabilizes AXIN	*TNKS*
**Negatively correlated genes with ZNF132**	
Prefoldin mediated transfer of substrate to CCT/TriC	*ACTG1, TUBA4A, TUBB3, PFDN2, TUBB4B, TUBB2A, TUBA1C, CCT7, TUBB6, CCT3, ACTB*
Transport of connexons to the plasma membrane	*TUBA4A, TUBB3, TUBB4B, TUBB2A, TUBA1C, GJB2, TUBB6*
Microtubule-dependent trafficking of connexons from Golgi to the plasma membrane	*TUBA4A, TUBB3, TUBB4B, TUBB2A, TUBA1C, TUBB6*
Neutrophil degranulation	*NAPRT, FABP5, TRAPPC1, PYCARD, ALDOA, S100A11, PA2G4, NME2, RHOG, S100A12, TUBB4B, JUP, SLPI, IMPDH1,*

	TOLLIP, PSMD13, PGAM1, ERP44, PSMB7, PKM, CST3, GSTP1, PKP1, PPIA, ATP6V1D, HMOX2, PYGL, ANXA2, PNP, DYNLL1
RHO GTPases activate IQGAPs	*ACTG1, TUBA4A, TUBB3, TUBB4B, TUBB2A, TUBA1C, TUBB6, ACTB*
Formation of the cornified envelope	*PKP3, PKP1, PI3, KRT6A, SPINK6, KRT6B, SPRR2G, KRT6C, CDSN, SPRR1B, KRT14, KRT16, KRT17, JUP, LCE3D, KLK8, LCE3E*
Gap junction trafficking	*GJB5, GJB6, GJB3, CLTB, TUBB6, ACTG1, TUBA4A, TUBB3, TUBB4B, TUBB2A, TUBA1C, GJB2, ACTB*
Formation of tubulin folding intermediates by CCT/TriC	*TUBA4A, TUBB3, TUBB4B, TUBB2A, TUBA1C, CCT7, TUBB6, CCT3*
Gap junction trafficking and regulation	*GJB5, GJB6, GJB3, CLTB, TUBB6, ACTG1, TUBA4A, TUBB3, TUBB4B, TUBB2A, TUBA1C, GJB2, ACTB*
Post-chaperonin tubulin folding pathway	*TUBA4A, TUBB3, TUBB4B, TUBB2A, TUBA1C, TUBB6*

Table S3. Involvement of *ZNFs* transcripts in cellular processes based on GSEA analysis in HNSCC patients. Normalized enrichment scores for GSEA analysis of MSigDB gene sets for oncogenic and hallmark genes sets in the group of patients with low and high expression levels of specified *ZNFs*. Only results set with $p \leq 0.05$ and $FDR \leq 0.25$ were shown. NES (normalized enrichment score), p-val (nominal p-value) and FDR q-val (false discovery rate).

**ZFP28 LOW**						
**HALLMARK**						
**Process**	**SIZE**	**ES**	**NES**	**NOM p-val**	FDR q-val	FWER p-val
HALLMARK_MYC_TARGETS_V1	188	-0.7033593	-1.9591562	0	0.032507837	0.031
ONCOGENIC						
Process	SIZE	ES	NES	NOM p-val	FDR q-val	FWER p-val
SINGH_KRAS_DEPENDENCY_SIGNATURE	20	-0.7966735	-1.7382913	0.01192843	0.09436689	0.245
RB_P107_DN.V1_DN	111	-0.41109267	-1.6093328	0.018404908	0.14880078	0.502
**ZFP28 HIGH**						
HALLMARK						
Process	SIZE	ES	NES	NOM p-val	FDR q-val	FWER p-val

No process				-		
ONCOGENIC						
Process	SIZE	ES	NES	NOM p-val	FDR q-val	FWER p-val
ESC_V6.5_UP_EARLY.V1_UP	153	0.41845626	1.7548374	0	0.062050827	0.245
BRCA1_DN.V1_DN	123	0.411691	1.68942	0	0.07300265	0.367
MTOR_UP.V1_DN	170	0.45409486	1.820618	0	0.08531365	0.139
BRCA1_DN.V1_UP	122	0.5070129	1.888495	0	0.09420856	0.075
IL2_UP.V1_DN	172	0.48583123	1.8270137	0	0.09847495	0.131
KRAS.PROSTATE_UP.V1_UP	126	0.5140462	1.9229596	0	0.112964265	0.045
STK33_DN	237	0.41059783	1.7932984	0.0019193857	0.063047	0.179
MTOR_UP.N4.V1_DN	154	0.53650737	1.7974787	0.0019379845	0.07784095	0.176
STK33_SKM_DN	235	0.41189855	1.7352247	0.0019417476	0.06588144	0.272
IL21_UP.V1_DN	166	0.4507301	1.7976047	0.001984127	0.09052824	0.176
BMI1_DN_MEL18_DN.V1_DN	137	0.5041968	1.878085	0.002008032	0.07285083	0.086
PRC1_BMI_UP.V1_UP	168	0.43189573	1.7296886	0.0020283975	0.06564677	0.284
IL15_UP.V1_DN	164	0.4607605	1.7774625	0.0020491802	0.061206225	0.201
KRAS.BREAST_UP.V1_DN	129	0.48597175	1.7937435	0.0020703934	0.07078621	0.179
EGFR_UP.V1_DN	166	0.3919127	1.5847212	0.0037664783	0.072305344	0.579
STK33_NOMO_DN	237	0.40316385	1.673443	0.0039138943	0.06845865	0.4
GCNP_SHH_UP_EARLY.V1_DN	157	0.39341584	1.6434138	0.006024096	0.06798053	0.466
CRX_NRL_DN.V1_UP	129	0.3669617	1.5494833	0.006024096	0.08243801	0.624
RAPA_EARLY_UP.V1_UP	153	0.37599713	1.5618075	0.00625	0.081019804	0.608
JNK_DN.V1_UP	170	0.43698993	1.6787323	0.0062761507	0.07056176	0.39
PTEN_DN.V2_DN	125	0.3897153	1.6050193	0.007797271	0.072676644	0.539
CTIP_DN.V1_UP	120	0.45687774	1.7085323	0.007952286	0.06766376	0.324
P53_DN.V2_DN	141	0.3707659	1.5477594	0.007984032	0.08199287	0.628
E2F3_UP.V1_UP	171	0.45879358	1.7036487	0.008016032	0.067471534	0.339
CYCLIN_D1_KE_.V1_DN	184	0.4035997	1.6490792	0.00814664	0.06719784	0.456

VEGF_A_UP.V1_UP	185	0.46470475	1.7566174	0.008213553	0.06612402	0.241
JNK_DN.V1_DN	174	0.45515335	1.781058	0.008230452	0.065064184	0.198
KRAS.AMP.LUNG_UP.V1_UP	130	0.4413422	1.6418837	0.008510638	0.06693244	0.474
NOTCH_DN.V1_UP	169	0.3896639	1.5877337	0.01004016	0.07531395	0.572
KRAS.KIDNEY_UP.V1_UP	136	0.5521734	1.7655439	0.01010101	0.065958135	0.226
CAHOY_ASTROCYTIC	97	0.40896687	1.586903	0.011741683	0.074115016	0.573
KRAS.600.LUNG.BREAST_UP.V1_DN	265	0.38493887	1.5926368	0.012072435	0.074088305	0.564
NOTCH_DN.V1_DN	170	0.40974233	1.6532688	0.012145749	0.06899748	0.44
PRC2_SUZ12_UP.V1_DN	169	0.37270784	1.5814753	0.0121951215	0.07277543	0.581
CAHOY_NEURONAL	94	0.4629674	1.6626521	0.012552301	0.06886005	0.422
ATF2_UP.V1_DN	170	0.46270254	1.6836009	0.013888889	0.07053963	0.378
ESC_V6.5_UP_LATE.V1_UP	170	0.43761042	1.6751012	0.0140280565	0.06981706	0.396
JAK2_DN.V1_UP	170	0.38216096	1.6006777	0.014403292	0.072148085	0.548
PTEN_DN.V1_UP	168	0.41533834	1.6022748	0.014522822	0.07268985	0.546
KRAS.AMP.LUNG_UP.V1_DN	126	0.43272254	1.6655899	0.014553014	0.06970211	0.415
PRC1_BMI_UP.V1_DN	171	0.38469452	1.6185759	0.015118791	0.06961899	0.519
ATF2_S_UP.V1_DN	176	0.47364253	1.7452166	0.015810277	0.064439915	0.259
CAHOY_OLIGODENDROCUTIC	85	0.3955319	1.5262989	0.015841585	0.08841384	0.654
KRAS.300_UP.V1_UP	136	0.47053823	1.6887871	0.016161617	0.0704136	0.369
RAPA_EARLY_UP.V1_DN	173	0.3436187	1.4682466	0.016460905	0.11274297	0.766
LTE2_UP.V1_UP	176	0.39246416	1.5957209	0.019157087	0.07350725	0.555
NRL_DN.V1_UP	129	0.34949672	1.460184	0.019607844	0.116726846	0.792
KRAS.600_UP.V1_UP	261	0.4583389	1.7088232	0.02020202	0.0710207	0.323
IL21_UP.V1_UP	172	0.37738997	1.5468907	0.020408163	0.08092316	0.628
BMI1_DN.V1_DN	128	0.4168698	1.6061801	0.020449897	0.07401484	0.539
MEL18_DN.V1_DN	137	0.45478103	1.6491336	0.021400778	0.06929777	0.456
DCA_UP.V1_DN	162	0.34401232	1.4968454	0.02173913	0.10153855	0.709

PTEN_DN.V1_DN	167	0.39030677	1.5519207	0.022088353	0.08270045	0.62
AKT_UP.V1_DN	179	0.43007925	1.6620604	0.022132797	0.06684079	0.424
CSR_LATE_UP.V1_DN	131	0.4277939	1.6382835	0.022267206	0.0667448	0.484
PRC2_SUZ12_UP.V1_UP	172	0.36924395	1.5611689	0.022312373	0.07971571	0.608
ATF2_S_UP.V1_UP	177	0.32519668	1.4505827	0.02330097	0.121853754	0.801
KRAS.50_UP.V1_UP	47	0.5456285	1.7176628	0.024489796	0.070453405	0.309
LEF1_UP.V1_UP	186	0.44475043	1.6381508	0.024539877	0.06502785	0.484
PKCA_DN.V1_DN	146	0.36159357	1.5272518	0.024896266	0.08938076	0.653
KRAS.600.LUNG.BREAST_UP.V1_UP	267	0.40791094	1.6299127	0.025641026	0.06769131	0.504
PIGF_UP.V1_DN	178	0.38522825	1.5867903	0.026804123	0.07262415	0.573
WNT_UP.V1_UP	169	0.3448856	1.4814402	0.027027028	0.1062937	0.739
P53_DN.V2_UP	142	0.421922	1.6280116	0.027600849	0.06675427	0.506
CTIP_DN.V1_DN	118	0.37865314	1.4894131	0.028056113	0.10304027	0.729
PRC2_EED_UP.V1_UP	174	0.35395628	1.4734133	0.030947777	0.11059456	0.753
ATF2_UP.V1_UP	181	0.34298852	1.4673829	0.033613447	0.111724384	0.767
SRC_UP.V1_UP	148	0.39200208	1.5065879	0.036659878	0.09646952	0.69
RELA_DN.V1_DN	127	0.38814613	1.5236872	0.038617887	0.08851045	0.659
KRAS.BREAST_UP.V1_UP	128	0.40197974	1.538174	0.04	0.08432543	0.64
CYCLIN_D1_UP.V1_DN	179	0.32745725	1.4013363	0.045908183	0.14873384	0.851
ERBB2_UP.V1_UP	179	0.3773266	1.5130033	0.04761905	0.09374291	0.68
DCA_UP.V1_UP	166	0.3245589	1.3904004	0.0499002	0.15251735	0.861
**ZNF132 LOW**						
HALLMARK						
Process	SIZE	ES	NES	NOM p-val	FDR q-val	FWER p-val
No process				-		
ONCOGENIC						
Process	SIZE	ES	NES	NOM p-val	FDR q-val	FWER p-val
No process				-		
**ZNF132 HIGH**						
HALLMARK						
Process	SIZE	ES	NES	NOM p-val	FDR q-val	FWER p-val

No process				-		
			ONCOGENIC			
Process	SIZE	ES	NES	NOM p-val	FDR q-val	FWER p-val
IL2_UP.V1_DN	172	0.48074335	1.8123108	0.0019305019	0.22845033	0.141
			**ZNF418 LOW**			
			HALLMARK			
Process	SIZE	ES	NES	NOM p-val	FDR q-val	FWER p-val
HALLMARK_MYC_TARGETS_V1	188	0.70635825	1.9595909	0.0019607844	0.024626149	0.034
HALLMARK_MYC_TARGETS_V2	58	0.6444798	1.6591418	0.06346154	0.24339326	0.359
			ONCOGENIC			
Process	SIZE	ES	NES	NOM p-val	FDR q-val	FWER p-val
SINGH_KRAS_DEPENDENCY_SIGNATURE	20	0.7439315	1.6562297	0.033398822	0.13443983	0.398
RB_P107_DN.V1_DN	111	0.3821421	1.5333258	0.03285421	0.17947969	0.641
			**ZNF418 HIGH**			
			HALLMARK			
Process	SIZE	ES	NES	NOM p-val	FDR q-val	FWER p-val
HALLMARK_KRAS_SIGNALING_UP	193	-0.5189731	-1.8128397	0.0019493178	0.2462509	0.126
HALLMARK_COAGULATION	136	-0.49198148	-1.767163	0.009633912	0.19013539	0.183
HALLMARK_IL2_STAT5_SIGNALING	194	-0.4797305	-1.759137	0.009823183	0.14186552	0.199
HALLMARK_COMPLEMENT	195	-0.46599516	-1.6740719	0.02702702 8	0.16059901	0.32
			ONCOGENIC			
Process	SIZE	ES	NES	NOM p-val	FDR q-val	FWER p-val
KRAS.PROSTATE_UP.V1_UP	126	-0.58782035	-2.2336104	0	0	0
KRAS.AMP.LUNG_UP.V1_UP	130	-0.54051894	-2.0606806	0	0.001913292	0.005
PTEN_DN.V1_UP	168	-0.5262224	-2.0632331	0	0.002232174	0.005
IL2_UP.V1_DN	172	-0.5585994	-2.1607866	0	0.002267471	0.002
KRAS.600_UP.V1_UP	261	-0.5446241	-2.0659223	0	0.002372522	0.004
IL15_UP.V1_DN	164	-0.5035248	-1.9823798	0	0.0025143845	0.021
KRAS.AMP.LUNG_UP.V1_DN	126	-0.546705	-2.1095316	0	0.0025592665	0.003

IL21_UP.V1_DN	166	-0.49548814	-2.0160117	0	0.0025682386	0.014
BRCA1_DN.V1_DN	123	-0.47888434	-1.9826748	0	0.0026820102	0.021
CTIP_DN.V1_UP	120	-0.54531586	-2.0308762	0	0.0027760696	0.011
BMI1_DN_MEL18_DN.V1_DN	137	-0.54153585	-2.0172582	0	0.0028250625	0.014
BRCA1_DN.V1_UP	122	-0.5335266	-1.9853559	0	0.0028735823	0.021
KRAS.BREAST_UP.V1_DN	129	-0.5525263	-2.069255	0	0.0029656522	0.004
ATF2_S_UP.V1_DN	176	-0.5485492	-1.9883087	0	0.0030135585	0.02
JNK_DN.V1_UP	170	-0.51217055	-2.0174675	0	0.0031389585	0.014
JNK_DN.V1_DN	174	-0.5023681	-1.9893346	0	0.003160669	0.019
PTEN_DN.V1_DN	167	-0.4804555	-1.9537499	0	0.0040733814	0.034
ATF2_UP.V1_DN	170	-0.5226324	-1.9297507	0	0.0047080237	0.044
PRC2_SUZ12_UP.V1_DN	169	-0.44569093	-1.9306606	0	0.004969581	0.044
DCA_UP.V1_UP	166	-0.44111717	-1.8949682	0	0.0060598324	0.065
NOTCH_DN.V1_DN	170	-0.47118273	-1.9080054	0	0.0061417907	0.058
CSR_LATE_UP.V1_DN	131	-0.490256	-1.8905959	0	0.0061941384	0.068
CYCLIN_D1_KE_.V1_DN	184	-0.4626264	-1.8963137	0	0.006205578	0.064
P53_DN.V2_DN	141	-0.44318217	-1.8760443	0	0.006249346	0.078
KRAS.600.LUNG.BREAST_UP.V1_DN	265	-0.45535234	-1.8769166	0	0.006433252	0.077
MEL18_DN.V1_DN	137	-0.51280814	-1.8598994	0	0.0064573325	0.093
BMI1_DN.V1_DN	128	-0.4769156	-1.8621953	0	0.006602323	0.089
KRAS.600.LUNG.BREAST_UP.V1_UP	267	-0.45851806	-1.878191	0	0.0066334005	0.076

PRC1_BMI_UP.V1_UP	168	-0.45566958	-1.8787928	0	0.006787498	0.076
ALK_DN.V1_UP	131	-0.44858015	-1.862391	0	0.0068153017	0.089
NOTCH_DN.V1_UP	169	-0.44470543	-1.8454466	0	0.007850302	0.114
RAPA_EARLY_UP.V1_UP	153	-0.43912217	-1.8330141	0	0.008585237	0.134
ATF2_S_UP.V1_UP	177	-0.4099953	-1.8184426	0	0.008942704	0.147
PKCA_DN.V1_DN	146	-0.42588738	-1.812706	0	0.008972448	0.154
MTOR_UP.N4.V1_DN	154	-0.5231579	-1.8121986	0	0.008976697	0.157
STK33_SKM_DN	235	-0.43082544	-1.8206153	0	0.009048403	0.147
PIGF_UP.V1_DN	178	-0.44184768	-1.8210858	0	0.009203777	0.146
GCNP_SHH_UP_EARLY.V1_DN	157	-0.4275579	-1.7971119	0	0.0094209695	0.174
ESC_V6.5_UP_EARLY.V1_UP	153	-0.4347706	-1.7997797	0	0.009439345	0.174
ESC_V6.5_UP_LATE.V1_UP	170	-0.4712934	-1.800405	0	0.009520494	0.173
CAHOY_OLIGODENDROCUTIC	85	-0.4540185	-1.7867582	0	0.009848738	0.183
CRX_DN.V1_UP	125	-0.41854867	-1.7739898	0	0.010278094	0.204
PRC2_EED_UP.V1_UP	174	-0.42936006	-1.7749729	0	0.01049131	0.201
PDGF_UP.V1_DN	117	-0.45574328	-1.7648411	0	0.011119064	0.214
JAK2_DN.V1_UP	170	-0.4131979	-1.74807	0	0.011834514	0.238
NRL_DN.V1_DN	121	-0.4069717	-1.7369152	0	0.012794922	0.252
STK33_DN	237	-0.3901516	-1.7097195	0	0.015277704	0.306
CRX_NRL_DN.V1_UP	129	-0.4063586	-1.7099057	0	0.015462178	0.306
ESC_J1_UP_EARLY.V1_UP	151	-0.39710423	-1.6955601	0	0.01747725	0.33

PRC1_BMI_UP.V1_DN	171	-0.39524233	-1.6849236	0	0.018238211	0.34
PRC2_SUZ12_UP.V1_UP	172	-0.41966596	-1.781849	0.0019305019	0.009906264	0.188
GCNP_SHH_UP_LATE.V1_DN	168	-0.3615609	-1.603944	0.001980198	0.032633055	0.504
ATM_DN.V1_UP	140	-0.4430142	-1.821271	0.001992032	0.009422914	0.146
CAHOY_ASTROCYTIC	97	-0.44907138	-1.7352251	0.002	0.012857592	0.256
MTOR_UP.V1_DN	170	-0.4528975	-1.843926	0.002016129	0.0076325005	0.116
KRAS.LUNG.BREAST_UP.V1_DN	132	-0.44205263	-1.714924	0.002016129	0.014939763	0.295
LTE2_UP.V1_UP	176	-0.42683712	-1.7637072	0.0020283975	0.011000088	0.215
AKT_UP.V1_DN	179	-0.47748542	-1.8606879	0.0020325202	0.0066007213	0.092
WNT_UP.V1_UP	169	-0.39803916	-1.7108196	0.0020408162	0.015608166	0.306
AKT_UP_MTOR_DN.V1_DN	177	-0.3926892	-1.7162569	0.0020618557	0.014812925	0.292
P53_DN.V2_UP	142	-0.4796643	-1.9070125	0.0020661156	0.006042478	0.059
STK33_NOMO_DN	237	-0.41628	-1.7516512	0.0020661156	0.011837807	0.231
YAP1_DN	40	-0.47512946	-1.6658838	0.00390625	0.020745037	0.377
ATF2_UP.V1_UP	181	-0.39781326	-1.6852853	0.003984064	0.018295987	0.339
DCA_UP.V1_DN	162	-0.38543215	-1.6451837	0.003984064	0.02503769	0.423
P53_DN.V1_DN	186	-0.49073446	-1.8404187	0.004	0.007796757	0.119
ATM_DN.V1_DN	140	-0.44353157	-1.7871188	0.004	0.009937515	0.181
KRAS.LUNG_UP.V1_UP	130	-0.44699708	-1.7470609	0.004	0.011721658	0.238
ALK_DN.V1_DN	128	-0.4361652	-1.7480787	0.004	0.012011149	0.238
KRAS.300_UP.V1_UP	136	-0.5224938	-1.9025966	0.004056795	0.0059404033	0.061

KRAS.KIDNEY_UP.V1_UP	136	-0.5694077	-1.8627278	0.004056795	0.006963199	0.088
CTIP_DN.V1_DN	118	-0.4513732	-1.8069801	0.0040650405	0.009011636	0.163
RELA_DN.V1_DN	127	-0.46369067	-1.8220394	0.0040650405	0.009528149	0.145
IL21_UP.V1_UP	172	-0.42654535	-1.7337606	0.004132231	0.012772453	0.256
WNT_UP.V1_DN	163	-0.42348513	-1.7570467	0.004149378	0.01142485	0.225
SNF5_DN.V1_UP	158	-0.49831802	-1.8134499	0.004158004	0.009095088	0.153
BCAT.100_UP.V1_UP	46	-0.48800933	-1.6691583	0.0042918455	0.020577064	0.374
NRL_DN.V1_UP	129	-0.38358018	-1.603741	0.0058252425	0.032307748	0.504
PTEN_DN.V2_DN	125	-0.39513987	-1.6186128	0.0059405942	0.02940702	0.469
KRAS.KIDNEY_UP.V1_DN	126	-0.4665823	-1.8453215	0.005988024	0.007632239	0.114
E2F1_UP.V1_DN	174	-0.4417965	-1.7285908	0.006134969	0.013194651	0.264
KRAS.BREAST_UP.V1_UP	128	-0.47430986	-1.8105708	0.0062240665	0.009002986	0.159
CAHOY_ASTROGLIAL	92	-0.5051626	-1.7940445	0.006263048	0.009618142	0.176
LEF1_UP.V1_UP	186	-0.49401978	-1.8104372	0.006289308	0.00886483	0.16
VEGF_A_UP.V1_UP	185	-0.4793994	-1.8289244	0.007889546	0.008873496	0.137
PTEN_DN.V2_UP	129	-0.43090525	-1.6312122	0.010330578	0.026899192	0.449
CRX_DN.V1_DN	128	-0.3943917	-1.5623249	0.010460251	0.040999338	0.581
KRAS.50_UP.V1_UP	47	-0.557791	-1.7746135	0.010504202	0.010338056	0.201
KRAS.600_UP.V1_DN	264	-0.413695	-1.6921346	0.011952192	0.017775951	0.335
CYCLIN_D1_UP.V1_DN	179	-0.36255866	-1.5663463	0.012170386	0.040135987	0.576
E2F3_UP.V1_UP	171	-0.44597954	-1.6856071	0.013435701	0.018448757	0.339

ERBB2_UP.V1_UP	179	-0.41327295	-1.6339794	0.013888889	0.026740182	0.441
ESC_J1_UP_LATE.V1_UP	175	-0.43856072	-1.6900861	0.014198783	0.017945576	0.337
EGFR_UP.V1_DN	166	-0.38333702	-1.5711657	0.016032064	0.039362743	0.568
LEF1_UP.V1_DN	177	-0.39249036	-1.5994887	0.016096579	0.033016857	0.515
TGFB_UP.V1_DN	179	-0.34654886	-1.496096	0.016359918	0.058768637	0.694
IL15_UP.V1_UP	171	-0.36973017	-1.5903349	0.018108651	0.034956086	0.535
PDGF_ERK_DN.V1_UP	132	-0.31495443	-1.4084008	0.018404908	0.091241226	0.83
KRAS.PROSTATE_UP.V1_DN	135	-0.4369884	-1.6348603	0.018789144	0.026860785	0.441
RPS14_DN.V1_UP	184	-0.5615499	-1.7861389	0.020576132	0.0097207865	0.183
RAPA_EARLY_UP.V1_DN	173	-0.3509905	-1.5225507	0.021032505	0.050456233	0.668
SRC_UP.V1_UP	148	-0.41064253	-1.5412781	0.021653544	0.046367165	0.631
CSR_EARLY_UP.V1_DN	111	-0.4124138	-1.5780567	0.02303263	0.037797373	0.556
RAF_UP.V1_DN	177	-0.40993452	-1.5448812	0.026156941	0.045764912	0.624
CAHOY_NEURONAL	94	-0.4488035	-1.6332852	0.027559055	0.02659221	0.442
RELA_DN.V1_UP	145	-0.3550513	-1.4845804	0.027888447	0.06248913	0.712
NFE2L2.V2	411	-0.36215115	-1.5801932	0.029354207	0.03746538	0.55
BCAT_GDS748_UP	46	-0.44279665	-1.5236131	0.032520324	0.0505429	0.663
PKCA_DN.V1_UP	150	-0.35619864	-1.5045141	0.032520324	0.056410078	0.688
IL2_UP.V1_UP	173	-0.38285378	-1.5565723	0.03305785	0.042757668	0.596
KRAS.300_UP.V1_DN	131	-0.39582765	-1.537856	0.035433073	0.046164736	0.64

RB_P130_DN.V1_UP	116	-0.41758975	-1.5385842	0.039014373	0.046780035	0.639
CYCLIN_D1_UP.V1_UP	178	-0.332474	-1.413482	0.046277665	0.08950229	0.821
KRAS.LUNG.BREAST_UP.V1_UP	135	-0.40545097	-1.5378803	0.04761905	0.046592187	0.64
SRC_UP.V1_DN	152	-0.30739236	-1.3628328	0.048387095	0.11347062	0.886
KRAS.DF.V1_DN	183	-0.33389047	-1.3887444	0.0499002	0.099742115	0.848
HOXA9_DN.V1_UP	171	-0.45063797	-1.5686555	0.05	0.039681017	0.571
**ZNF426 LOW**						
HALLMARK						
Process	SIZE	ES	NES	NOM p-val	FDR q-val	FWER p-val
HALLMARK_DNA_REPAIR	140	0.5191116	1.8323119	0.012048192	0.07233542	0.125
HALLMARK_OXIDATIVE_PHOSPHORYLATION	182	0.6202922	1.8700818	0.014141414	0.09219361	0.089
ONCOGENIC						
Process	SIZE	ES	NES	NOM p-val	FDR q-val	FWER p-val
CAMP_UP.V1_UP	180	0.40318057	1.7084215	0.0019157088	0.17119835	0.302
**ZNF426 HIGH**						
HALLMARK						
Process	SIZE	ES	NES	NOM p-val	FDR q-val	FWER p-val
HALLMARK_TGF_BETA_SIGNALING	54	-0.60554296	-1.8469702	0.004149378	0.16819642	0.092
HALLMARK_PROTEIN_SECRETION	95	-0.5346111	-1.7968848	0.006085193	0.13189258	0.138
HALLMARK_UV_RESPONSE_DN	137	-0.5330411	-1.7297308	0.008510638	0.11955917	0.225
HALLMARK_ANDROGEN_RESPONSE	96	-0.47598895	-1.7401007	0.010121457	0.1433816	0.212
HALLMARK_ESTROGEN_RESPONSE_EARLY	192	-0.4221948	-1.6895446	0.014373717	0.12728834	0.281
ONCOGENIC						
Process	SIZE	ES	NES	NOM p-val	FDR q-val	FWER p-val
TBK1.DF_DN	257	-0.57272047	-1.9589195	0	0.013526973	0.027
EIF4E_DN	90	-0.6262681	-2.0373917	0	0.014703409	0.011

PIGF_UP.V1_UP	177	-0.58485496	-1.9663792	0.003992016	0.017775398	0.023
**ZNF540 LOW**						
HALLMARK						
Process	SIZE	ES	NES	NOM p-val	FDR q-val	FWER p-val
No process				-		
ONCOGENIC						
Process	SIZE	ES	NES	NOM p-val	FDR q-val	FWER p-val
CSR_EARLY_UP.V1_UP	136	0.5592234	1.8651284	0.0038387715	0.06822576	0.091
SINGH_KRAS_DEPENDENCY_SIGNATURE	20	0.77383715	1.7358071	0.02734375	0.13370766	0.248
**ZNF540 HIGH**						
HALLMARK						
Process	SIZE	ES	NES	NOM p-val	FDR q-val	FWER p-val
No process				-		
ONCOGENIC						
Process	SIZE	ES	NES	NOM p-val	FDR q-val	FWER p-val
KRAS.BREAST_UP.V1_DN	129	-0.48146132	-1.7783498	0	0.09449233	0.185
KRAS.PROSTATE_UP.V1_UP	126	-0.47695076	-1.8134872	0	0.10699397	0.147
PRC2_SUZ12_UP.V1_DN	169	-0.40205407	-1.7224718	0	0.113517255	0.274
IL2_UP.V1_DN	172	-0.48304752	-1.8390303	0	0.115910225	0.117
CTIP_DN.V1_UP	120	-0.51356244	-1.8974354	0.0020283975	0.10731563	0.064
KRAS.AMP.LUNG_UP.V1_DN	126	-0.4467045	-1.7455815	0.003816794	0.11496834	0.231
MTOR_UP.N4.V1_DN	154	-0.512408	-1.7972623	0.004115226	0.09601281	0.166
CRX_DN.V1_UP	125	-0.37504476	-1.6064051	0.0057471264	0.14348851	0.485
DCA_UP.V1_DN	162	-0.35928047	-1.5646865	0.005905512	0.109942	0.574
KRAS.AMP.LUNG_UP.V1_UP	130	-0.45628554	-1.6898639	0.005952381	0.11085421	0.319
BRCA1_DN.V1_UP	122	-0.47231078	-1.7444317	0.006134969	0.09975463	0.231

IL15_UP.V1_DN	164	-0.43752256	-1.6933297	0.006198347	0.117857724	0.312
P53_DN.V2_DN	141	-0.38830706	-1.636611	0.0062761507	0.15717748	0.423
ATF2_S_UP.V1_UP	177	-0.3585978	-1.5911301	0.007984032	0.13876121	0.515
BMI1_DN_MEL18_DN.V1_DN	137	-0.46183267	-1.7192783	0.008179959	0.10366639	0.279
STK33_DN	237	-0.36303443	-1.6009351	0.008179959	0.13472173	0.493
PTEN_DN.V1_UP	168	-0.40361953	-1.5676091	0.0102459015	0.11487645	0.566
BRCA1_DN.V1_DN	123	-0.3892924	-1.6127898	0.010288066	0.15470336	0.473
PRC1_BMI_UP.V1_DN	171	-0.3623015	-1.5305016	0.01192843	0.12328486	0.648
KRAS.600.LUNG.BREAST_UP.V1_DN	265	-0.39462203	-1.6206548	0.012	0.1523793	0.454
IL21_UP.V1_DN	166	-0.39948338	-1.6069379	0.014	0.15124331	0.482
PRC2_SUZ12_UP.V1_UP	172	-0.36811048	-1.5815599	0.0140280565	0.12937869	0.527
ESC_V6.5_UP_EARLY.V1_UP	153	-0.36161226	-1.5080565	0.015904572	0.12503007	0.688
CSR_EARLY_UP.V1_DN	111	-0.4237799	-1.6317422	0.016460905	0.15188019	0.435
STK33_SKM_DN	235	-0.3678678	-1.5844793	0.016632017	0.13277137	0.526
SRC_UP.V1_UP	148	-0.42013294	-1.5690321	0.017612524	0.12205873	0.562
CRX_NRL_DN.V1_UP	129	-0.37110302	-1.5689805	0.017964073	0.117779285	0.562
NOTCH_DN.V1_DN	170	-0.38993746	-1.5779254	0.018329939	0.122393265	0.534
JAK2_DN.V1_UP	170	-0.36246854	-1.4954052	0.019723866	0.13069381	0.713
MTOR_UP.V1_DN	170	-0.39138243	-1.6027552	0.02053388	0.13898748	0.488
JNK_DN.V1_UP	170	-0.40097693	-1.5703297	0.020618556	0.12515377	0.557

STK33_NOMO_DN	237	-0.36331493	-1.5206835	0.022821577	0.1278755	0.668
E2F3_UP.V1_UP	171	-0.4123802	-1.5503093	0.027184466	0.11100326	0.608
LEF1_UP.V1_DN	177	-0.3852115	-1.5596665	0.028397566	0.110290155	0.581
PRC1_BMI_UP.V1_UP	168	-0.361633	-1.4804169	0.028397566	0.13344479	0.738
BMI1_DN.V1_DN	128	-0.4102482	-1.5902628	0.02892562	0.13300468	0.516
CAHOY_OLIGODENDROCUTIC	85	-0.36707383	-1.4753858	0.030425964	0.13191949	0.743
RAPA_EARLY_UP.V1_UP	153	-0.35685802	-1.4766347	0.03168317	0.13372901	0.742
P53_DN.V2_UP	142	-0.39356774	-1.5785142	0.032258064	0.12714235	0.534
PTEN_DN.V1_DN	167	-0.3780826	-1.5146405	0.035643563	0.12573686	0.679
ATM_DN.V1_UP	140	-0.3551041	-1.4652463	0.03629032	0.13487558	0.759
DCA_UP.V1_UP	166	-0.32990485	-1.4171975	0.036363635	0.1565429	0.82
ALK_DN.V1_DN	128	-0.37353215	-1.482125	0.036893204	0.13498974	0.735
KRAS.LUNG.BREAST_UP.V1_DN	132	-0.37819216	-1.4714079	0.03984064	0.13236107	0.748
PIGF_UP.V1_DN	178	-0.36524495	-1.4974983	0.041749503	0.13134493	0.708
GCNP_SHH_UP_EARLY.V1_DN	157	-0.34490037	-1.436735	0.044303797	0.1480858	0.803
KRAS.300_UP.V1_DN	131	-0.38741973	-1.483671	0.045454547	0.13633	0.73
JNK_DN.V1_DN	174	-0.38636258	-1.5199273	0.047325104	0.12496605	0.67
**ZNF880 LOW**						
HALLMARK						
Process	SIZE	ES	NES	NOM p-val	FDR q-val	FWER p-val
HALLMARK_MYC_TARGETS_V1	188	0.7553346	2.1108708	0	0.0019721505	0.005
ONCOGENIC						
Process	SIZE	ES	NES	NOM p-val	FDR q-val	FWER p-val

SINGH_KRAS_DEPENDENCY_SIGNATURE	20	0.7726228	1.7392375	0.022132797	0.0793264	0.25
**ZNF880 HIGH**						
HALLMARK						
Process	SIZE	ES	NES	NOM p-val	FDR q-val	FWER p-val
HALLMARK_KRAS_SIGNALING_UP	193	-0.53431296	-1.8807771	0.0019417476	0.13277923	0.073
HALLMARK_IL2_STAT5_SIGNALING	194	-0.492885	-1.8228027	0.008016032	0.08579951	0.128
HALLMARK_COAGULATION	136	-0.50367165	-1.8331504	0.009505703	0.11030985	0.114
HALLMARK_WNT_BETA_CATENIN_SIGNALING	42	-0.48259205	-1.6514124	0.017578125	0.19889294	0.353
HALLMARK_KRAS_SIGNALING_DN	188	-0.3903482	-1.6159383	0.02053388	0.15808839	0.408
HALLMARK_APICAL_SURFACE	43	-0.43622726	-1.5511712	0.022680413	0.18224429	0.515
HALLMARK_UV_RESPONSE_DN	137	-0.50507885	-1.6149762	0.032945737	0.14073399	0.408
HALLMARK_BILE_ACID_METABOLISM	112	-0.3690332	-1.4684645	0.036659878	0.20230177	0.674
HALLMARK_EPITHELIAL_MESENCHYMAL_TRANSITION	194	-0.6100307	-1.671884	0.047244094	0.21426366	0.324
ONCOGENIC						
Process	SIZE	ES	NES	NOM p-val	FDR q-val	FWER p-val
BRCA1_DN.V1_UP	122	-0.5570718	-2.0580592	0	0.0020800577	0.006
JNK_DN.V1_UP	170	-0.5336213	-2.1285303	0	0.002087807	0.002
IL21_UP.V1_DN	166	-0.51926875	-2.0645976	0	0.0023379507	0.005
KRAS.600_UP.V1_UP	261	-0.5205863	-1.966695	0	0.0026885504	0.019
KRAS.PROSTATE_UP.V1_UP	126	-0.56847614	-2.0996742	0	0.0027082842	0.004
BMI1_DN.V1_DN	128	-0.50684017	-1.9754233	0	0.0027929503	0.019
JNK_DN.V1_DN	174	-0.4984375	-1.976734	0	0.0030077924	0.019
PTEN_DN.V1_UP	168	-0.50598216	-1.9572101	0	0.0031016576	0.024
KRAS.KIDNEY_UP.V1_UP	136	-0.5996813	-1.958878	0	0.0031779842	0.023

P53_DN.V1_DN	186	-0.53364855	-1.9778731	0	0.0032584418	0.019
IL15_UP.V1_DN	164	-0.51011443	-2.0009875	0	0.003524591	0.018
MEL18_DN.V1_DN	137	-0.5422305	-1.9807446	0	0.0035546639	0.019
CYCLIN_D1_KE_.V1_DN	184	-0.4780042	-1.982091	0	0.0039101304	0.019
BMI1_DN_MEL18_DN.V1_DN	137	-0.5401704	-2.0023165	0	0.0039651645	0.018
NOTCH_DN.V1_DN	170	-0.53131735	-2.142779	0	0.004175614	0.002
ATF2_UP.V1_DN	170	-0.5211129	-1.9302804	0	0.0042912527	0.035
KRAS.300_UP.V1_UP	136	-0.52791625	-1.9322002	0	0.0044714063	0.035
ATF2_S_UP.V1_DN	176	-0.5439721	-2.003248	0	0.004531617	0.018
STK33_SKM_DN	235	-0.45205534	-1.9254154	0	0.004575907	0.038
CTIP_DN.V1_UP	120	-0.5206223	-1.9216428	0	0.004628704	0.04
IL2_UP.V1_DN	172	-0.54043347	-2.009067	0	0.0050823945	0.018
LTE2_UP.V1_UP	176	-0.45892027	-1.89521	0	0.0055797915	0.048
MTOR_UP.N4.V1_DN	154	-0.53565717	-1.8682472	0	0.0056409477	0.069
P53_DN.V2_DN	141	-0.44605988	-1.8683933	0	0.005778914	0.069
BRCA1_DN.V1_DN	123	-0.46360722	-1.8856051	0	0.005828718	0.059
KRAS.50_UP.V1_UP	47	-0.58948886	-1.8632555	0	0.0058599375	0.076
CAHOY_OLIGODENDROCUTIC	85	-0.4713739	-1.8782787	0	0.0059171855	0.064
ATM_DN.V1_DN	140	-0.46260473	-1.8887779	0	0.006066877	0.056
VEGF_A_UP.V1_UP	185	-0.5057899	-1.8879248	0	0.006167337	0.057
KRAS.600.LUNG.BREAST_UP.V1_DN	265	-0.44763425	-1.8423011	0	0.006297128	0.094

PRC1_BMI_UP.V1_UP	168	-0.4602045	-1.890119	0	0.006319664	0.056
JAK2_DN.V1_UP	170	-0.44131655	-1.8521793	0	0.0063638673	0.087
ESC_V6.5_UP_EARLY.V1_UP	153	-0.43832377	-1.8425586	0	0.0064076055	0.094
MTOR_UP.V1_DN	170	-0.46054733	-1.844177	0	0.0065484666	0.094
AKT_UP.V1_DN	179	-0.47791442	-1.8311363	0	0.0069455355	0.107
PKCA_DN.V1_DN	146	-0.4262535	-1.826998	0	0.0069882134	0.11
PRC2_SUZ12_UP.V1_DN	169	-0.43229005	-1.8212489	0	0.007667074	0.122
GCNP_SHH_UP_EARLY.V1_DN	157	-0.43469486	-1.8087583	0	0.008180328	0.137
KRAS.AMP.LUNG_UP.V1_DN	126	-0.46731302	-1.7980953	0	0.0085180355	0.147
KRAS.AMP.LUNG_UP.V1_UP	130	-0.48403093	-1.7991425	0	0.008654803	0.147
RAPA_EARLY_UP.V1_UP	153	-0.43607247	-1.7919617	0	0.00900596	0.157
PRC2_EED_UP.V1_UP	174	-0.42479056	-1.7809367	0	0.009064169	0.177
KRAS.BREAST_UP.V1_DN	129	-0.48750436	-1.7870405	0	0.009092369	0.168
CAHOY_ASTROCYTIC	97	-0.45094764	-1.7721968	0	0.009581484	0.19
CYCLIN_D1_UP.V1_DN	179	-0.3866293	-1.6980233	0	0.017255528	0.326
ATF2_S_UP.V1_UP	177	-0.3779935	-1.6573681	0	0.022855176	0.405
KRAS.600.LUNG.BREAST_UP.V1_UP	267	-0.44168696	-1.7998439	0.0019157088	0.008810786	0.147
AKT_UP_MTOR_DN.V1_DN	177	-0.37567648	-1.6380923	0.0019379845	0.02564906	0.441
ESC_J1_UP_LATE.V1_UP	175	-0.47640935	-1.8625531	0.0019417476	0.0056971614	0.076
CTIP_DN.V1_DN	118	-0.45270532	-1.817208	0.0019455253	0.007675021	0.126

KRAS.BREAST_UP.V1_UP	128	-0.49138674	-1.8869253	0.0019493178	0.005979215	0.058
PRC2_SUZ12_UP.V1_UP	172	-0.39345	-1.6650878	0.0019493178	0.021565003	0.394
PDGF_UP.V1_DN	117	-0.44655433	-1.788049	0.001953125	0.009129695	0.166
PTEN_DN.V1_DN	167	-0.47171223	-1.872992	0.0019607844	0.0055950647	0.067
ESC_V6.5_UP_LATE.V1_UP	170	-0.47877994	-1.813443	0.0019880715	0.007824713	0.128
STK33_NOMO_DN	237	-0.4136329	-1.7405137	0.001992032	0.01249526	0.24
NOTCH_DN.V1_UP	169	-0.41443375	-1.7255309	0.002	0.013890607	0.274
STK33_DN	237	-0.39182377	-1.713288	0.0020120724	0.01491797	0.288
LEF1_UP.V1_UP	186	-0.51297694	-1.8779308	0.002020202	0.0057199458	0.064
E2F1_UP.V1_DN	174	-0.42144293	-1.6897892	0.002020202	0.018517975	0.343
DCA_UP.V1_UP	166	-0.4088643	-1.7393749	0.0020283975	0.0124191595	0.241
KRAS.KIDNEY_UP.V1_DN	126	-0.44764072	-1.7195729	0.0020491802	0.014284342	0.281
RELA_DN.V1_DN	127	-0.47289214	-1.8196473	0.002057613	0.007642161	0.123
IL21_UP.V1_UP	172	-0.43361217	-1.7476187	0.0020618557	0.011944198	0.231
ATM_DN.V1_UP	140	-0.4321151	-1.7387314	0.0020876827	0.012274184	0.242
CAHOY_ASTROGLIAL	92	-0.5239279	-1.8736614	0.003984064	0.0057402295	0.067
PTEN_DN.V2_UP	129	-0.46778616	-1.7916532	0.0040241447	0.008836037	0.157
KRAS.PROSTATE_UP.V1_DN	135	-0.47534904	-1.7864103	0.0040241447	0.008954919	0.169
LEF1_UP.V1_DN	177	-0.4180833	-1.6887565	0.0040241447	0.018235663	0.345
PRC1_BMI_UP.V1_DN	171	-0.38765362	-1.6065522	0.0040241447	0.03068809	0.496

CRX_DN.V1_UP	125	-0.39805472	-1.689496	0.004056795	0.01831965 3	0.345
P53_DN.V2_UP	142	-0.452817	-1.7841852	0.005905512	0.009054973	0.174
KRAS.600_UP.V1_DN	264	-0.4198844	-1.7122804	0.0059405942	0.014829157	0.291
IL15_UP.V1_UP	171	-0.36926493	-1.5682822	0.006012024	0.03826376	0.57
ESC_V6.5_UP_EARLY.V1_DN	153	-0.4833999	-1.803128	0.0060362173	0.008608512	0.142
WNT_UP.V1_DN	163	-0.42527688	-1.7544483	0.0060728746	0.011233805	0.218
EGFR_UP.V1_DN	166	-0.40838	-1.6716326	0.0060975607	0.020747665	0.383
CRX_NRL_DN.V1_UP	129	-0.3736479	-1.5742764	0.0061728396	0.0368991	0.549
CSR_LATE_UP.V1_DN	131	-0.45841214	-1.7828397	0.007827789	0.009072429	0.176
GCNP_SHH_UP_LATE.V1_DN	168	-0.33212557	-1.4565773	0.009881423	0.06613243	0.769
KRAS.LUNG.BREAST_UP.V1_DN	132	-0.4208828	-1.64033	0.009940358	0.025494892	0.438
ESC_J1_UP_EARLY.V1_UP	151	-0.38175395	-1.6222422	0.00996016	0.027840758	0.469
RAF_UP.V1_DN	177	-0.44678962	-1.7229601	0.0113852	0.014208747	0.278
NRL_DN.V1_UP	129	-0.37117139	-1.55595	0.0116731515	0.040011402	0.587
KRAS.LUNG_UP.V1_UP	130	-0.4630544	-1.7793784	0.011952192	0.009058625	0.18
RAPA_EARLY_UP.V1_DN	173	-0.36384967	-1.5795484	0.012072435	0.036613017	0.544
PKCA_DN.V1_UP	150	-0.36280525	-1.5431333	0.012121212	0.04195279	0.613
RPS14_DN.V1_UP	184	-0.5755643	-1.9073397	0.013592233	0.004849815	0.042
WNT_UP.V1_UP	169	-0.37155545	-1.5699593	0.01417004	0.038031798	0.565
SNF5_DN.V1_DN	147	-0.40873495	-1.6342388	0.015779093	0.026170108	0.448
RELA_DN.V1_UP	145	-0.37134862	-1.541634	0.015968064	0.041987497	0.617

ERBB2_UP.V1_UP	179	-0.40779245	-1.6321173	0.017964073	0.026348103	0.451
BCAT.100_UP.V1_DN	28	-0.4691471	-1.5784229	0.017964073	0.03647975	0.544
ALK_DN.V1_DN	128	-0.40317044	-1.586379	0.018072288	0.03500745	0.525
PTEN_DN.V2_DN	125	-0.38241166	-1.5513586	0.019120459	0.040214244	0.594
ATF2_UP.V1_UP	181	-0.3634727	-1.5213069	0.01980198	0.04589369	0.658
DCA_UP.V1_DN	162	-0.33696416	-1.4625524	0.01996008	0.0644632	0.754
SNF5_DN.V1_UP	158	-0.4820875	-1.7539169	0.020746889	0.011089715	0.218
CRX_DN.V1_DN	128	-0.37215453	-1.5050715	0.021568628	0.049879286	0.682
ALK_DN.V1_UP	131	-0.37871242	-1.550308	0.021956088	0.040053047	0.597
KRAS.300_UP.V1_DN	131	-0.4152209	-1.5910677	0.022177419	0.03410655	0.521
STK33_SKM_UP	238	-0.4503159	-1.6263297	0.02244898	0.027279435	0.46
HOXA9_DN.V1_UP	171	-0.48529422	-1.6817813	0.022774328	0.019099293	0.357
E2F3_UP.V1_UP	171	-0.41815433	-1.5769879	0.024253732	0.036506344	0.545
RAF_UP.V1_UP	182	-0.38838157	-1.5095226	0.026	0.04897479	0.678
KRAS.LUNG.BREAST_UP.V1_UP	135	-0.43092874	-1.6563247	0.026666667	0.022731023	0.407
CAHOY_NEURONAL	94	-0.45999014	-1.655503	0.027888447	0.022648733	0.408
NFE2L2.V2	411	-0.35505763	-1.5227377	0.030181086	0.04578266	0.656
SRC_UP.V1_UP	148	-0.41054234	-1.5533906	0.030710172	0.040495623	0.593
P53_DN.V1_UP	184	-0.3905705	-1.5518457	0.033663366	0.04050672	0.594
NRL_DN.V1_DN	121	-0.33900133	-1.458594	0.036885247	0.06566516	0.76

BCAT_GDS748_UP	46	-0.45797893	-1.5607423	0.037037037	0.039323255	0.577
CAMP_UP.V1_DN	186	-0.40553352	-1.5637769	0.037623763	0.039070915	0.574
BCAT.100_UP.V1_UP	46	-0.45253932	-1.524421	0.039175257	0.046237078	0.656
KRAS.DF.V1_UP	182	-0.38395998	-1.5589074	0.04183267	0.03953119	0.579
KRAS.DF.V1_DN	183	-0.3370468	-1.4191353	0.04191617	0.081076354	0.824
TGFB_UP.V1_DN	179	-0.32163996	-1.3935256	0.042510122	0.09110349	0.861
SRC_UP.V1_DN	152	-0.3125242	-1.4091281	0.04347826	0.08529137	0.841
PIGF_UP.V1_DN	178	-0.37361568	-1.500715	0.044989776	0.0514219	0.694
CYCLIN_D1_UP.V1_UP	178	-0.34060344	-1.433372	0.049281314	0.07528666	0.804
PRC2_EZH2_UP.V1_UP	180	-0.39766768	-1.5231307	0.05	0.046152893	0.656

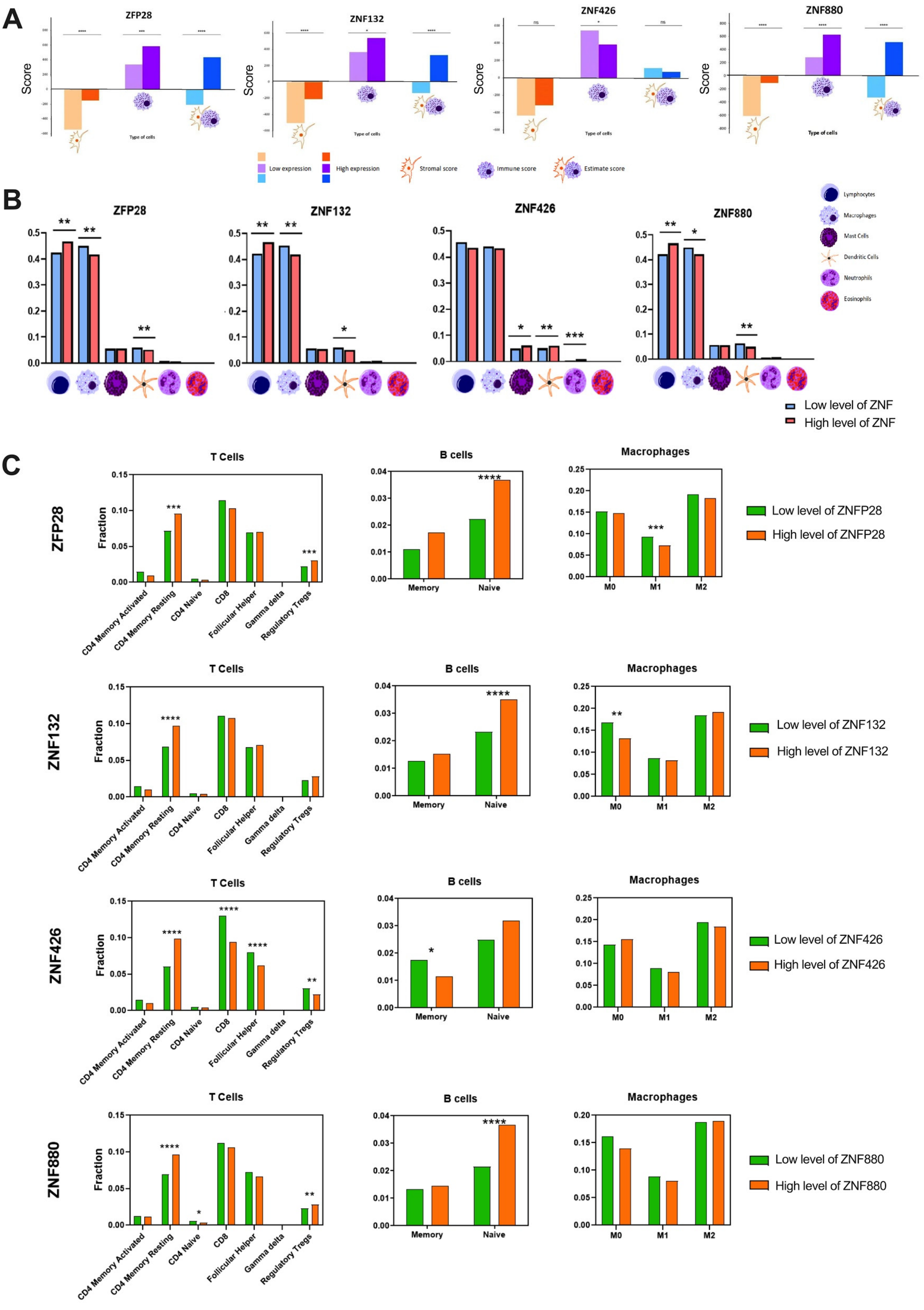

Figure S3. The immunological profile of HNSCC patients depends on the low and high level of *ZFP28*, *ZNF132*, *ZNF426* and *ZNF880* transcripts. A) Stromal, immune, and ESTIMATE scores; B) Infiltration of specific immune cells in tumor samples; C) Differences in the fraction of T cells, B cells

and macrophages; *ns* - not significant; * $p \leq 0.05$; ** $p \leq 0.01$; *** $p \leq 0.001$; **** $p \leq 0.0001$ considered as significant.

Table S4. Expression level of *ZNF540* depending on clinical-pathological parameters in HNSCC patients from GSE65858; Mann-Whitney U test or one-way ANOVA, $p < 0.05$ considered as significant.

**Parameter**	**Group**	**Mean ± SEM**	**P-val**	**Cases**
Age	<60	6.448 ± 0.01302	0.3211	157
	>60	6.449± 0.01304		113
Gender	Female	6.469 ± 0.02326	0.2616	47
	Male	6.444 ± 0.01016		223
Alcohol	Positive	6.444 ± 0.009569	0.2698	239
	Negative	6.487± 0.03356		31
Smoking	Positive	6.439 ± 0.009893	0.0418	222
	Negative	6.491 ± 0.02489		48
UICC Stage	I + II	6.431 ± 0.01561	0.8075	55
	III + IV	6.453 ± 0.01100		215
T stage	T1 + T2	6.454 ± 0.01377	0.4456	115
	T3 + T4	6.445 ± 0.01264		155
N stage	N0	6.432 ± 0.01287	0.6297	94
	N1+N2+N3	6.457 ± 0.01251		176
Localization	Oral cavity	6.423 ± 0.01353	> 0.05	83
	Hypopharynx	6.404 ± 0.01454		33
	Larynx	6.416 ± 0.01448		48
	Oropharynx	6.498 ± 0.01893		102
